# Influence of P-wave oblique incidence on seismic response of helical piles in soft soil sites

**DOI:** 10.1038/s41598-025-92808-w

**Published:** 2025-06-13

**Authors:** Hang Cen, Hui-yue Wang, De-long Huang, Jian-rong Xu, Sha-sha Yu, Chang-lu Xu, Zhong-ling Zong, Wen Zhou, Zi-yuan Huang

**Affiliations:** 1 School of Civil and Ocean Engineering, Jiangsu Ocean University, Lianyungang, 222005 China; 2 Logistics and Infrastructure Administrative Office, Jiangsu Ocean University, Lianyungang, 222005 China; 3 Earthquake Administration of Hainan Province, Haikou, 570203 China; 4 School of Civil Engineering, Harbin Institute of Technology, Harbin, 150090 China

**Keywords:** Helical pile, Marine soft soil sites, Oblique incidence of P-wave, Viscous-spring boundaries, Equivalent seismic nodal force, Vertical displacement, Dynamic *p-y* curves, Natural hazards, Civil engineering

## Abstract

In regions susceptible to earthquakes, an increasing number of building structures are employing helical piles as their foundational system due to their commendable seismic performance. This paper investigates the vertical displacements of the helical pile-soil model, dynamic *p-y* curves, and seismic subsidence of helical piles in marine soft soil sites under seismic motions, considering the effects of various types of seismic waves, seismic intensity, angle of incidence, and the number of helical blades. The results demonstrate that the vertical displacement of double-blade helical piles is smaller than that of single-blade helical piles. Furthermore, the vertical displacement of helical pile-soil systems is influenced by the type of seismic wave, seismic intensity, and angle of incident. Moreover, the seismic subsidence of helical piles is significantly influenced by the peak ground acceleration and the frequency of the seismic wave, both of which are related to the angle of incident. Finally, this paper rectifies the *p-y* curve of soft soil in the API specification based on the angle of incidence. The conclusions of this study provide a basis for the seismic design of helical piles in marine soft soil sites.

## Introduction

In civil engineering, precast concrete piles and grouted concrete piles continue to serve as reliable and economical foundation solutions for a wide range of engineering applications. However, in seismic-prone areas and specific geological environments, such as marine soft soils, these traditional pile foundations may present certain limitations. For instance, structures utilizing these foundations experienced significant damage in liquefied sites during the Niigata Earthquake in Japan^1^, the Fukushima Earthquake^2^, and the Kaikoura Earthquake in New Zealand^3^. In contrast, helical piles, comprising a steel shaft with one or more helical blades, are receiving considerable attention in marine engineering and seismic-prone regions because of their ease of installation, cost-effectiveness, and superior performance in challenging soft soil environments^4,5^. The adaptability of helical piles is particularly pronounced in marine soft soil environments. For instance, offshore lighthouses, marine floating structures, and various marine structures have heightened requirements for earthquake and wind resistance^6^. Elsawy et al.^7^ conducted full-scale shaking table tests to investigate the effects of varying earthquake frequencies, seismic intensities, pile geometries, installation methods, and other factors on the natural frequency of helical piles. The results indicated that the natural frequency of helical piles is a critical factor influencing their seismic response, and their lateral response aligns with that of conventional piles. The addition of blades can enhance the dynamic load-bearing performance of helical piles to a certain extent. Orang et al.^8^ replicated the study conducted by Elsawy et al.^7^ on a non-full scale and ultimately replicated the original test results. Furthermore, their findings highlighted that the number of helical blades exerts boundary effects on the seismic response. Shahbazi et al.^9^ investigated the damping properties of single and grouped helical piles in dense sand, revealing that single helical piles possess higher damping ratios than grouped helical piles, although the latter consume more energy. Additionally, the findings predict the range of damping expected from helical piles under actual earthquake conditions, aiding in the selection of suitable geometry and connection types. Fayez et al.^10^ investigated the seismic performance of helical piles in dry sand through shaking table tests, conclusively indicating that the influence of varying seismic frequencies must be considered in seismic design, and that soil reactions on the sides of single helical piles are more nonlinear compared to those on grouped helical piles. Fayez et al.^11^, utilizing full-scale shaking table tests, investigated how depth of embedment, cross-sectional dimensions, installation method, number of helical blades, and shape of the helical piles affect the helical pile-soil interaction under dry sand conditions, finding that these factors significantly affect the natural frequency of the helical pile-soil structure, elucidating the dynamics of pile-soil interaction. Moreover, these studies not only affirmed the superior seismic performance of helical piles under earthquake conditions in comparison to conventional piles, but also indicated a predominant scholarly focus on non-dynamic factors affecting seismic response, such as the natural frequency of helical piles, soil reaction force, lateral displacement, and bending moment of the pile. In contrast, less attention is afforded to dynamic response factors, such as earthquake-induced settlement (seismic subsidence) and dynamic *p-y* curves.

The dynamic *p-y* curve is one of the methods used to study pile-soil interaction. This method presents several advantages, including the ability to circumvent the need for selecting soil constitutive models, offer a clear conceptual framework, simplifying modeling, and facilitating testing^12^. Furthermore, Elsawy et al.^13^ investigated the seismic response of helical piles in dry sand, emphasizing the influence of loading frequency, peak ground acceleration (*PGA*), installation method, number of helical blades, geometry, and type of coupling on the dynamic *p-y* curves. The findings indicated that the installation method, number of helical blades, and geometric factors did not significantly affect the primary branch of the dynamic *p-y* curves. Further investigation into the dynamic *p-y* curve of helical piles is essential.

Marine soft soils are characterized by low strength, high compressibility, and high sensitivity^14,15^. China’s extensive coastline, which stretches from the northern part of Liaoning Province to the southern regions of Guangdong, Guangxi, and Hainan Provinces, encompasses many areas where marine soft soils are prevalent, which are also high-intensity seismic zones. Under specific conditions, geological hazards associated with marine soft soils, such as surface collapse and subsidence, can be readily induced by ground shaking, additional loading, or the presence of internal water^16^. Currently, researchers are investigating the issues related to seismic subsidence and settlement in soft soil foundations. Wang et al.^17^ examined the impact of changes in principal stress rotation on the deformation of soft soil during seismic events. The results indicated that rotation of the principal stress axis increased the susceptibility of soft soil. Furthermore, helical piles not only significantly mitigate the damage settlements cause to buildings^18^, but also reduce uplift damage to underground pipeline networks and tunnels when incorporated into their design^19^. Consequently, conducting a more comprehensive investigation into the seismic subsidence response of helical piles in soft soil sites is essential.

Recent advancements have been made in the study of seismic responses of underground structures subjected to oblique incidence conditions of seismic waves. Liu et al.^20^ introduced a method for seismic wave input that focuses on structure-foundation dynamic interaction, treating seismic waves as equivalent seismic nodal forces. This approach has been demonstrated to possess high accuracy. Du et al.^21^ developed a formulation for 3D SV wave equivalent seismic nodal forces associated with a viscous-spring artificial boundary; the feasibility of this formulation was confirmed through the analysis of seismic response patterns in a rock tunnel section. Mamoon and Ahmad^22^ analyzed the seismic response of a single pile under oblique incidence of P and S waves, discovering that obliquely incident waves produce larger displacements compared to vertically incident waves. Utilizing the hybrid boundary element method, Mamoon and Banerjee^23^ examined the dynamic response of both single and grouped piles to vertical and oblique seismic wave incidences. The authors concluded that obliquely incident waves elicit a more pronounced seismic response than those that are vertically incident. Subsequently, Álamo et al.^24^ employed a combined three-dimensional frequency domain boundary element-finite element (BEM–FEM) model to study the effects of pile-soil-structure interaction in response to obliquely incident shear waves. Wang et al.^25^ formulated an analytical model to assess the seismic response of large-span bridges subjected to oblique P waves, revealing that the incident angle significantly affects the model. Medina et al.^26^ and He et al.^27^ further confirmed the substantial effect of oblique incidence of seismic waves on piles. Currently, the majority of research on the seismic resistance of helical pile foundations assumes vertical incidence of seismic waves. However, investigations into seismic damage have shown that seismic waves from near-field strong earthquakes, originating from shallow depths, often strike obliquely at certain angles, leading to damage to foundation structures; thus, a more comprehensive investigation is warranted. In addition, compared with SV wave, P wave, as a compression wave, has a more significant effect on seismic settlement and vertical displacement due to its ability to cause compression and expansion of the foundation and the surrounding soil. Therefore, P wave is chosen as one of the main research focuses in this paper.

Dynamic analysis of underground structures frequently necessitates consideration of both static and dynamic soil equilibrium issues. Thus, when analyzing the dynamic response of subsurface structures using the finite element method, it is imperative to transition from static to dynamic problem consideration. However, prior research has predominantly focused on the soil’s static equilibrium while often neglecting its dynamic equilibrium. The insufficient understanding of the principle of artificial boundary transition in static-dynamic analysis leads to numerical results that deviate from reality, thereby affecting the accuracy of dynamic analysis results. This paper addresses the aforementioned issues by considering the conditions for artificial boundary transformation^28–31^. Additionally, although it has been shown that increasing the number of helical pile blades can enhance the seismic performance of helical piles to a certain extent, this study further investigates helical pile-soil interactions as well as the seismic performance were further investigated under varying blade numbers, *PGA*s, seismic waves types, and incidence angles, in conjunction with visco-elastic artificial boundaries and equivalent seismic loads. The specific research concepts are outlined below:

1. A three-dimensional finite element model of the helical pile-soil system is established using ABAQUS software. Furthermore, a static-dynamic joint analysis of the underground structure is performed using an enhanced coupled boundary simulation method. Subsequently, equivalent seismic nodal forces are applied to each boundary node.
2. Various influencing factors, such as incident angle, *PGA*, number of helical blades, and seismic wave frequency, are analyzed to determine the vertical displacement of the helical pile-soil model under earthquake motions and the evolution of the dynamic *p-y* curve.
3. The study explores the variation in seismic entrapment of helical piles under various operational conditions, and adjusts the *p-y* curve for helical piles based on the incident angle as specified by the API.

## Methodology

### Viscous-spring artificial boundary

In the context of artificial boundaries, the viscous-spring boundary is recognized for its exceptional stability, being free from issues related to low-frequency drift and high-frequency oscillation instabilities. This method is characterized by its conceptual clarity, ease of implementation, and widespread application in structural calculations. The general formulation of this method is expressed as follows^32^:

1 $$\sigma_{li} \left( t \right) = - K_{li} u_{li} \left( t \right) - C_{li} \frac{{\partial u_{li} }}{\partial t}\left( t \right)$$

where $l$ is the node number of the artificial boundary; $t$ is time; $\sigma_{li}$, $u_{li}$, $\partial u_{li} /\partial t$ are the stresses, displacements and velocities of the node in the calculation direction, respectively; $K_{li}$, $C_{li}$ are the parameters of the spring and damping elements of the viscous-spring artificial boundary of the node in the calculation direction, respectively.

Deeks and Randolph^33^, together with Liu and Lv^20^, introduced a two-dimensional viscous-spring artificial boundary that demonstrates the capability to mimic the elastic recovery properties of the semi-infinite medium beyond the artificial boundary, exhibiting notable high and low-frequency stability. Du et al.^32^ developed three-dimensional viscous-spring boundary conditions, utilizing the empirical superposition of plane waves and far-field scattering waves, to accurately reflect out-source wave propagation. The damping and spring parameters of the viscous-spring boundary, in both tangential and normal directions, are specified as follows:

2 $$K_{ln} = A_{l} \frac{1}{1 + A}\frac{\lambda + 2G}{r}$$

3 $$C_{ln} = A_{l} B\rho c_{p}$$

4 $$K_{lt} = A_{l} \frac{1}{1 + A}\frac{G}{r}$$

5 $$C_{it} = A_{l} B\rho c_{s}$$

where $n$ and $t$ are the normal and tangential directions of the boundary surface which the node belongs to, respectively; $K$ and $C$ are the spring and damping parameters, respectively; $\lambda$, $G$ and $\rho$ are the Lame constant, shear modulus and mass density, respectively; $A$ is the stiffness modified coefficient, which is recommended to take the value of 0.8; $B$ is the damping modified coefficient, which is recommended to take the value of 1.1; $c_{p} = \left( {(\lambda + 2G)/\rho } \right)^{1/2}$ and $c_{s} = \left( {G/\rho } \right)^{1/2}$ denote the P-wave and S-wave wave speeds, respectively; $A_{l}$ is the control area of the node; $r$ is the distance from the node to the center of the structure.

### Equivalent seismic nodal forces at artificial boundaries

Currently, two primary methods are available for applying seismic loads. The first method entails converting seismic waves into equivalent loads on artificial boundaries, which facilitates the replication of earthquake motions within the near-field computational domain without hindering the absorption of scattering waves by these boundaries^34–38^. The second method, referred to as the fluctuation method and proposed by Liu et al.^39^, involves seismic wave input on viscous-spring artificial boundaries. This approach employs seismic response analysis of the free-field model to derive equivalent seismic nodal forces, providing high computational accuracy, albeit with more complex implementation steps in practical applications. In this study, seismic loads will be applied to each viscous-spring artificial boundary condition, and its general form delineated as follows^39^:

6 $$F_{li} = L_{l} \left( {\sigma_{li}^{F} + K_{li} u_{li}^{F} + C_{li} \frac{{\partial u_{li}^{F} }}{\partial t}} \right)$$

where $F_{li}$ is the equivalent seismic nodal force; $L_{l} = \sum\limits_{e = 1}^{N} {L_{le} }$ is the range of influence of viscous-spring boundary stress at the artificial boundary node $l$, where $N$ is the number of elements adjacent to node $l$, and $L_{le}$ is the range of influence of viscous-spring boundary stress on element *e*; $u_{li}^{F}$,$\partial u_{li}^{F} /\partial t$,$\sigma_{li}^{F}$ are the displacements, velocities, and surface stresses of the site response at the artificial boundary, respectively; and $K_{li}$,$C_{li}$ are the viscous-spring artificial boundary of spring and damping parameters, which can be computed from Eqs. (2–5).

**Fig. 1:**
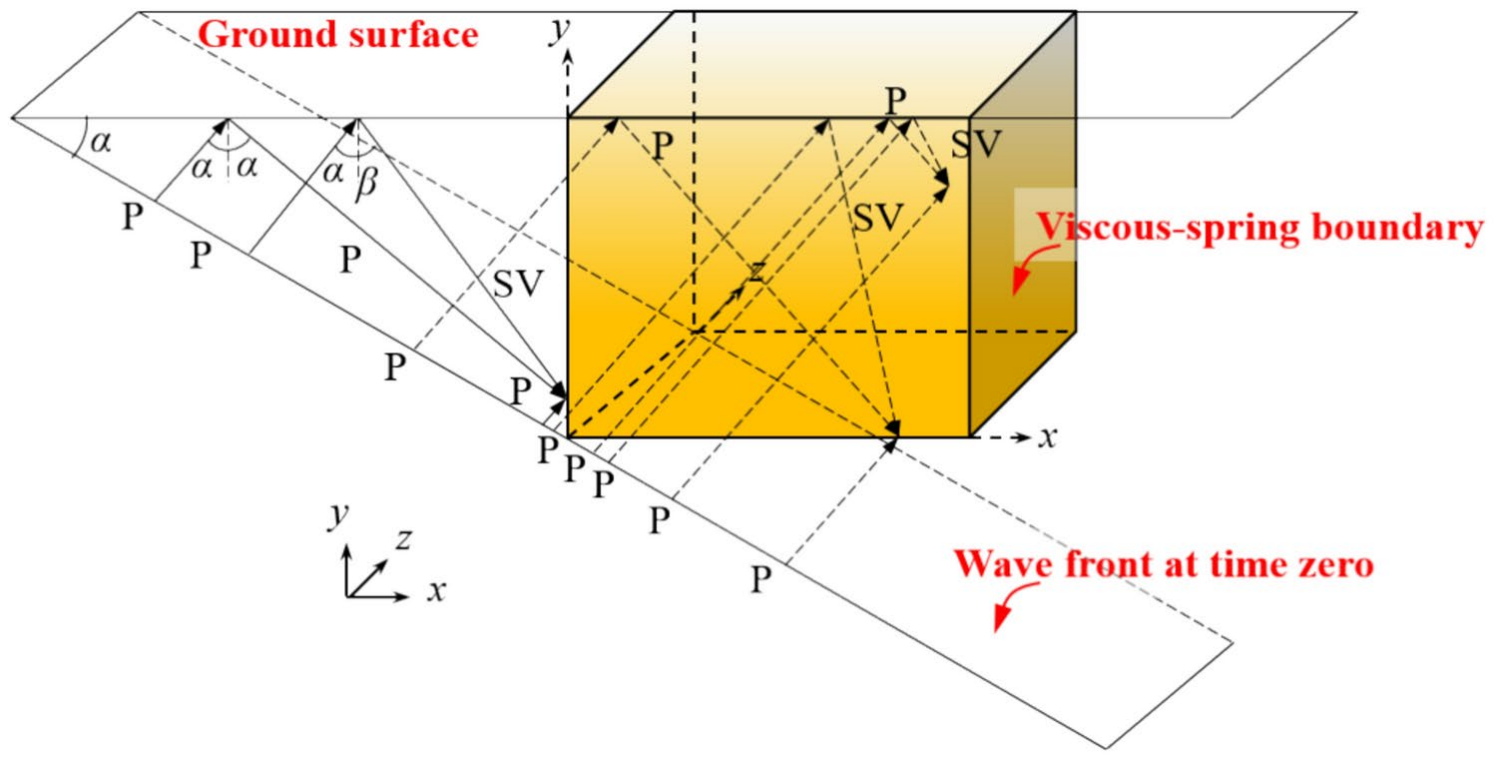
3D model P-wave oblique incidence.

Figure 1 illustrates the oblique incidence of the P wave from the wavefront to the artificial boundary at time zero. The P wave is reflected into two waveforms: one P wave with a specified incident angle and one SV wave with a distinct incident angle, where the vibration direction of the SV wave is perpendicular to that of the P wave. To the left of and within the artificial boundary, the oblique incidence of P waves predominantly comprises ground-reflected P waves, SV waves, and directly incident P waves, while directly below the artificial boundary, it primarily consists of directly incident P waves. Considering the ground’s equilibrium and continuity, it is feasible to deduce the general form of free-field motion and stresses at the nodes^40,41^:

7 $$\left\{ \begin{gathered} u_{lx}^{f} (t) = u_{0} (t - \Delta t_{1} )\sin \alpha + B_{1} u_{0} (t - \Delta t_{2} )\sin \alpha + B_{2} u_{0} (t - \Delta t_{3} )\cos \beta \hfill \\ u_{ly}^{f} (t) = u_{0} (t - \Delta t_{1} )\cos \alpha - B_{1} u_{0} (t - \Delta t_{2} )\cos \alpha + B_{2} u_{0} (t - \Delta t_{3} )\sin \beta \hfill \\ u_{lz}^{f} (t) = 0 \hfill \\ \frac{{\partial u_{lx}^{f} }}{\partial t}(t) = \frac{{\partial u_{0} (t - \Delta t_{1} )}}{\partial t}\sin \alpha + B_{1} \frac{{\partial u_{0} (t - \Delta t_{2} )}}{\partial t}\sin \alpha + B_{2} \frac{{\partial u_{0} (t - \Delta t_{3} )}}{\partial t}\cos \beta \hfill \\ \frac{{\partial u_{ly}^{f} (t)}}{\partial t} = \frac{{\partial u_{0} (t - \Delta t_{1} )}}{\partial t}\cos \alpha - B_{1} \frac{{\partial u_{0} (t - \Delta t_{2} )}}{\partial t}\cos \alpha + B_{2} \frac{{\partial u_{0} (t - \Delta t_{3} )}}{\partial t}\sin \beta \hfill \\ \frac{{\partial u_{lz}^{f} (t)}}{\partial t} = 0 \hfill \\ \end{gathered} \right.$$

where *B*_1_ and *B*_2_ are the amplitude ratios of the reflected P wave to the incident P wave and the reflected SV wave to the incident P wave, respectively; $\Delta t$ is the time taken for the wave to travel from the incident surface to the node *l* at time 0; and $\beta$ is the angle of reflection of the SV wave. Where the parameters $\Delta t$, $B_{1}$, $B_{2}$ and $\beta$ are denoted as:

8 $$B_{1} = \frac{{c_{s}^{2} \sin 2\alpha \sin 2\beta - c_{p}^{2} \cos^{2} 2\beta }}{{c_{s}^{2} \sin 2\alpha \sin 2\beta + c_{p}^{2} \cos^{2} 2\beta }}$$

9 $$B_{2} = \frac{{2c_{s} c_{p} \sin 2\alpha \cos 2\beta }}{{c_{s}^{2} \sin 2\alpha \sin 2\beta + c_{p}^{2} \cos^{2} 2\beta }}$$

10 $$\beta = \arcsin \left( {\frac{{c_{s} \sin \alpha }}{{c_{p} }}} \right)$$

11 $$\Delta t_{1} = \frac{x\sin \alpha + y\cos \alpha }{{c_{p} }}$$

12 $$\Delta t_{2} = \frac{{x\sin \alpha + (2L_{y} - y)\cos \alpha }}{{c_{p} }}$$

13 $$\Delta t_{3} = \frac{{L_{y} - y}}{{c_{s} \cos \beta }} + \frac{{x\sin \alpha + L_{y} \cos \alpha - (L_{y} - y)\tan \beta \sin \alpha }}{{c_{p} }}$$

where $L_{y}$ is the height of the model. The stress at node $l$ is:

14 $$\left\{ \begin{gathered} \sigma_{lx}^{f} (t) = S_{1} \left( {\frac{{\partial u_{0} (t - \Delta t_{1} )}}{\partial t} + S_{2} B_{1} \frac{{\partial u_{0} (t - \Delta t_{2} )}}{\partial t}} \right) + S_{3} B_{2} \frac{{\partial u_{0} (t - \Delta t_{3} )}}{\partial t} \hfill \\ \sigma_{ly}^{f} (t) = S_{4} \left( {\frac{{\partial u_{0} (t - \Delta t_{1} )}}{\partial t} + S_{5} B_{1} \frac{{\partial u_{0} (t - \Delta t_{2} )}}{\partial t}} \right) + S_{6} B_{2} u_{0} (t - \Delta t_{3} ) \hfill \\ \sigma_{lz}^{f} (t) = S_{7} \left( {\frac{{\partial u_{0} (t - \Delta t_{1} )}}{\partial t} + B_{1} \frac{{\partial u_{0} (t - \Delta t_{2} )}}{\partial t}} \right) \hfill \\ \end{gathered} \right.$$

where $S_{1}$ and $S_{7}$ are the boundary-related variables. At the bottom boundary, these variables are $S_{1} = G\sin 2\alpha /c_{p}$ $S_{2} = - 1$, $S_{3} = - G\cos 2\beta /c_{s}$, $S_{4} = \left( {\lambda + 2G\cos^{2} \alpha } \right)/c_{p}$, $S_{5} = 1$, $S_{6} = - G\sin 2\beta /c_{s}$ and $S_{7} = 0$; at the left boundary, these variables are $S_{1} = \left( {\lambda + 2G\sin^{2} 2\alpha } \right)/c_{p}$, $S_{2} = 1$, $S_{3} = G\sin 2\beta /c_{s}$, $S_{4} = G\sin 2\alpha /c_{p}$, $S_{5} = - 1$, $S_{6} = - G\cos 2\beta /c_{s}$; at the right boundary, the stress variables are the same as those at the left boundary, with the opposite direction; similarly the front and rear boundaries are set up in a manner similar to that of the left and right boundaries.

### Simulation verification

**Fig. 2:**
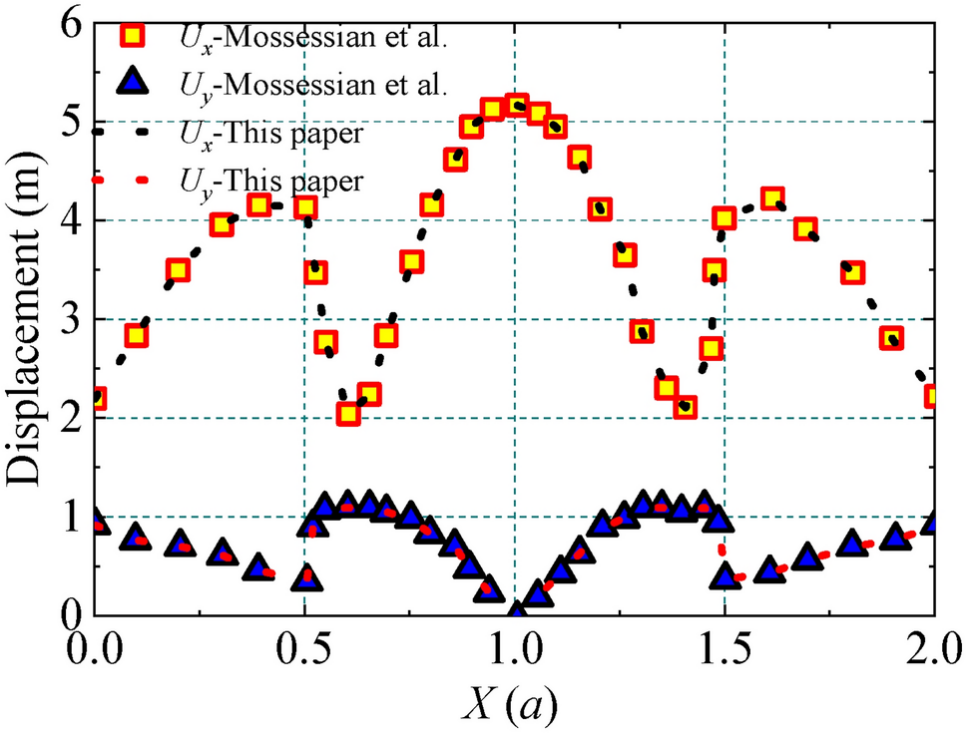
Simulation verification with Mossessian.

To verify the accuracy of the research method presented in this paper, the correctness of the method used in this paper for solving the seismic response of a three-dimensional depressed terrain site is verified by comparing it with the frequency domain results of the plane P-wave scattering by a depressed hemisphere in the literature^42^, where the Poisson’s ratio is 1/3 and the damping ratio is 0.005. The Poisson’s ratio is 1/3, the damping ratio is 0.005, the dimensionless frequency *η* = 1.0, and the incident plane P-wave is vertical. The comparative analysis results are shown in Fig. 2, where *U*_*x*_ and *U*_y_ represent the horizontal and vertical displacement amplitudes, respectively, and it can be seen that the results of this paper are in good agreement, which verifies the accuracy of this paper’s method, and illustrates the correctness of using vicious-spring artificial boundaries and the equivalent nodal forces in the calculations, along with the corresponding computational methods.

### Model establishment of helical pile and soil

#### Finite element model

**Fig. 3:**
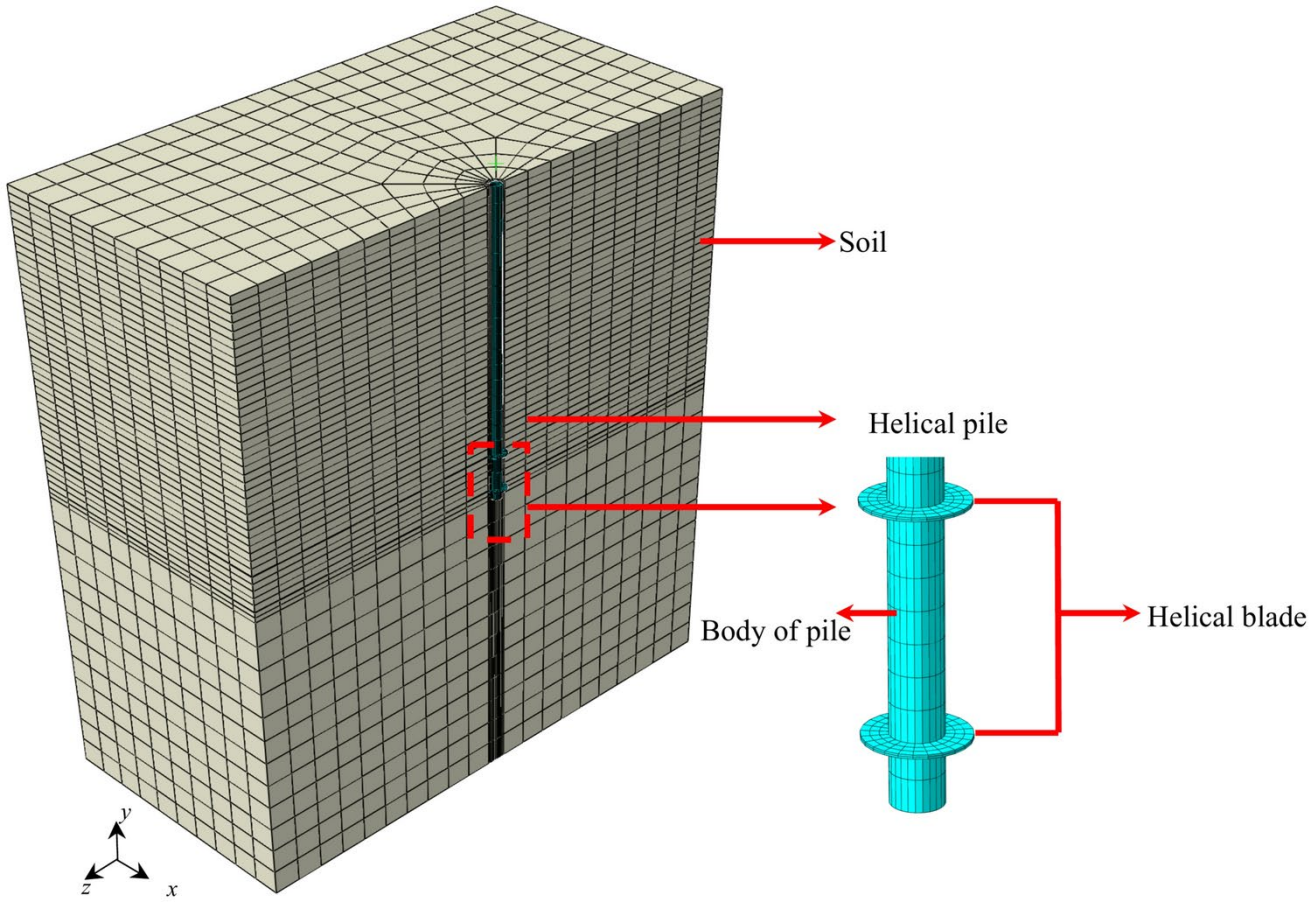
Helical pile-soil finite element model.

To investigate the vertical displacement, seismic subsidence, and the dynamic *p-y* curve of the helical pile during the oblique incidence of the P wave, a three-dimensional finite element model of the helical pile-soil system is initially established. This model primarily consists of the soil and the helical pile, which is positioned in the central region of the soil and has an embedment depth of 3 m, as shown in Fig. 3. The direction of the *x*-axis is the direction of the incident seismic wave, and the *y*-axis is the vertical direction. Since there is an inclination angle in the helical blade of the helical pile in the actual project; therefore, the helical blade is modeled as a plane disc to simplify the analysis. This modification reduces calculation errors compared to the case of having an inclination angle, allowing us to neglect its influence^42^.

The soil finite element model consists of solid elements with dimensions of 5 × 5 × 6 m. To ensure an acceptable mesh quality for the soil model, it is necessary to partition the model into distinct areas. This partitioning facilitates the subsequent division of each region into elements using a structured meshing approach. To enhance the accuracy of the pile model and improve the understanding of the interaction between the pile and soil, the soil surrounding the pile is subdivided. Additionally, to guarantee that the soil mesh size remains smaller than the pile mesh, the soil within the height range of the pile is also subdivided. This approach improved convergence during the calculation process. The final soil model consists of 29,196 solid elements. This paper also highlights the impact of grid size on the seismic wave’s distortion, with the grid size being determined according to the following formula^44^:

15 $$\frac{{V_{S} }}{{12f_{\max } }} \le h_{\max } \le \frac{{V_{S} }}{{6f_{\max } }}$$

where $V_{s}$ is the shear wave velocity of the soil layer; $f_{\max }$ is the cut-off frequency, which is generally selected as the highest frequency in the seismic wave spectrum.

**Fig. 4:**
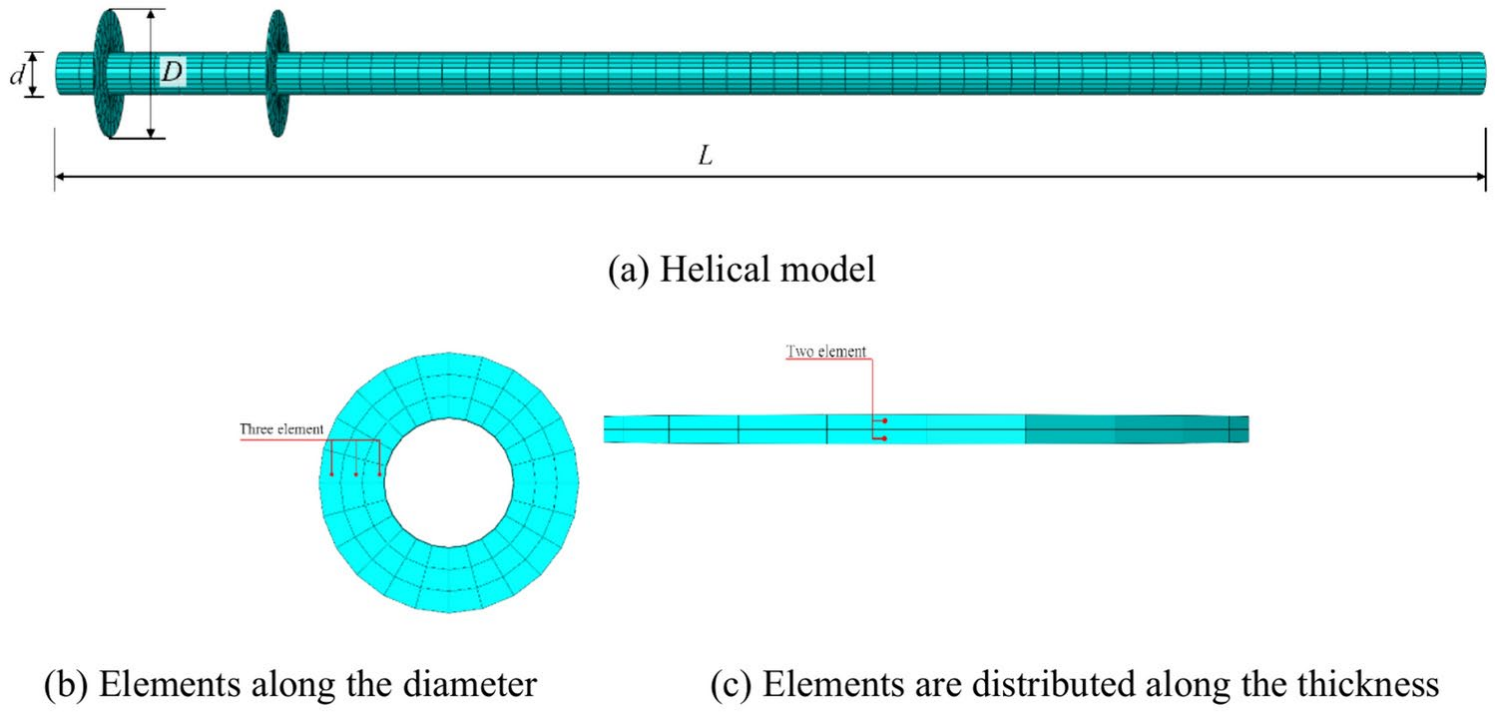
Helical pile model and meshing.

Figure 4a depicts the solid finite element model of the helical pile. The diameter of the pile measures 0.89 m, and the wall thickness is 6 mm. The diameter of the helical blade measures 1.78 m, which is twice the diameter of the pile, and the wall thickness remains 6 mm. The distance between two helical blades equals twice the diameter of the pile. The pile model is further divided into zones, allowing for its subdivision into elements based on a structured grid. The helical blade of the pile is subdivided into two grid elements along the blade’s thickness and three grid elements across the blade’s diameter. The pile is subdivided into a total of 1440 elements, as depicted in Fig. 4b and c.

#### Materials and contact

**Fig. 5:**
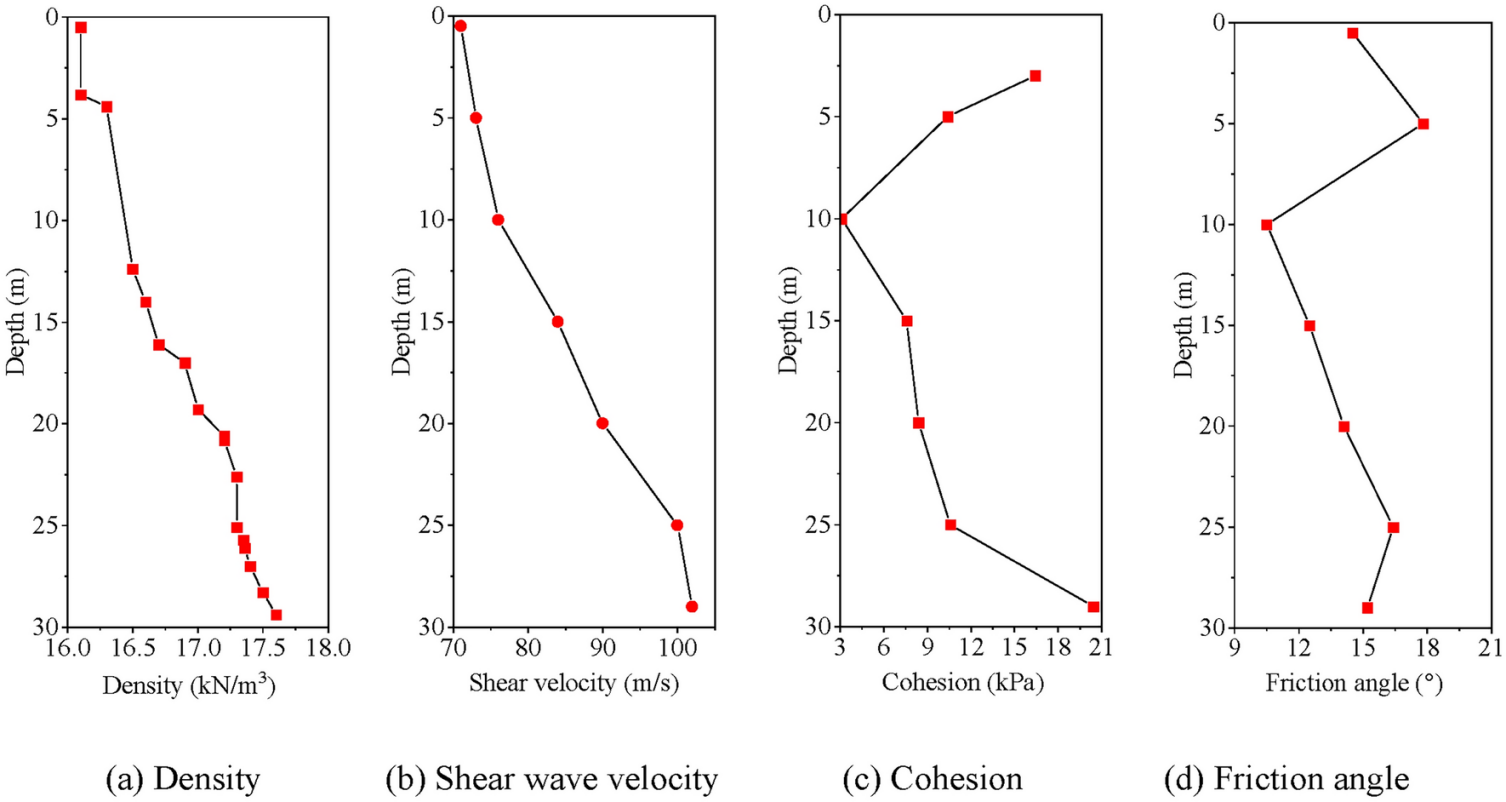
Soil indicators.

This model utilizes Lianyungang marine soft soil as its soil type. According to the literature^45^, the marine soft soil layer in the Lianyungang area is divided into two layers based on geological investigations. The upper layer consists of clay in a soft-plastic state, exhibiting moderately high compressibility and a thickness ranging from 0 to 2.5 m. The lower layer is made up of grey and dark grey fluid-plastic and partly soft-state silty clay, characterized by low bearing capacity, high sensitivity, and insufficient drainage and consolidation. The mechanical parameters of each soil layer are depicted in Fig. 5. To mitigate the impact of soil property variations in the buried area on the helical pile’s seismic subsidence, constructing a seismic subsidence analysis model for the homogenized soil layer is essential. Furthermore, an interpolation method is employed to homogenize the soil layer to a depth of 5 m, as depicted in Table 1. The soil employs an elastoplastic constitutive model using Mohr–Coulomb as the damage criterion, excellently responding to the mechanical properties of soft soil. This paper primarily addresses two aspects: the helical pile’s seismic subsidence and the dynamic *p-y* curve. Regarding the dynamic *p-y* curve, “*p*” denotes the lateral load, which is the soil’s reaction force on the pile, and “*y*” signifies the relative displacement between the pile and soil at the pile’s side in the far field^46^. Previous studies indicate that the load–displacement response of transversely loaded piles remains unaffected by the Mohr–Coulomb model^47^. Consequently, many researchers have utilized the Mohr–Coulomb model to investigate the damage criterion for laterally loaded piles^48–51^. Concerning seismic subsidence, researchers have developed elastic–plastic constitutive models that incorporate the Mohr–Coulomb damage criterion, utilizing the softened model analysis method^52–54^.

**Table 1:** Soil constitutive model and contact model parameter table.

Material	Density/kg·m^−3^	Young’s modulus/MPa	Poisson’s ratio	Friction angle/°	Cohesive force/kPa	Coefficient of friction
Soil	1890	2	0.30	15	15	0.1

For the helical piles, Q235 mild steel is utilized. The stress–strain relationship in steel can be categorized into the elastic stage and the plastic stage. In this study, Hooke’s law describes the elastic phase, and flow theory characterizes the plastic phase. According to flow theory, a singular yield surface exists, and incremental relationships between stress and plastic strain are established, alongside the associated reinforcement law. This study utilizes the following expression for the steel’s elastic–plastic constitutive model:

16 $$\sigma_{s} = \left\{ {\begin{array}{*{20}l} {E_{s} \varepsilon_{s} } \hfill & {\varepsilon_{s} \le \varepsilon_{e} } \hfill \\ { - A\varepsilon_{s}^{2} + B\varepsilon_{s} + C} \hfill & {\varepsilon_{e} < \varepsilon_{s} \le \varepsilon_{e1} } \hfill \\ {f_{y} } \hfill & {\varepsilon_{e1} < \varepsilon_{s} \le \varepsilon_{e2} } \hfill \\ {f_{y} \left[ {1 + 0.6\frac{{\varepsilon_{s} - \varepsilon_{e2} }}{{\varepsilon_{e3} - \varepsilon_{e2} }}} \right]} \hfill & {\varepsilon_{e2} < \varepsilon_{s} \le \varepsilon_{e3} } \hfill \\ {1.6f_{y} } \hfill & {\varepsilon_{s} > \varepsilon_{e3} } \hfill \\ \end{array} } \right.$$

Strain in elastic stage:

17 $$\varepsilon_{e} = 0.8f_{y} /E_{s}$$

Strain in elastic–plastic stage:

18 $$\varepsilon_{e1} = 1.5\varepsilon_{e}$$

Strain in plastic stage:

19 $$\varepsilon_{e2} = 10\varepsilon_{e1}$$

Strain in the reinforcement of stage:

20 $$\varepsilon_{e3} = 100\varepsilon_{e1}$$

21 $$A = 0.2f_{y} /\left( {\varepsilon_{e1} - \varepsilon_{e} } \right)^{2}$$

22 $$B = 2A\varepsilon_{e1}$$

23 $$C = 0.8f_{y} + A\varepsilon_{e}^{2} - B\varepsilon_{e}$$

where $f_{y}$ is the yield strength of the steel; $\varepsilon_{e}$ is the maximum strain before the steel enters the elastic–plastic stage. The parameters of elastic and plastic stages of steel are shown in Table 2.

**Table 2:** Steel elasticity stage parameters.

Material	Young’s modulus/GPa	Poisson’s ratio	Density/kg·m^−3^	Plastic stress	Plastic strain
Q235	212	0.288	7800	235.312594	0
				255.39565	0.023754793
				426.015094	0.12287613
				462.025962	0.203852574

Face-to-face contact, defined by tangential and normal behaviors as its contact qualities, is selected as the type of contact. The tangential behavior is defined as Coulomb friction, and the normal behavior is described as hard contact. The expression for Coulomb friction type behavior is:

24 $$\tau_{{{\text{crit}}}} = \mu p$$

where $\tau_{{{\text{crit}}}}$ is the critical stress; $\mu$ the friction coefficient; $p$ the forward contact pressure at the contact surface. In the model, the friction coefficient of the pile-soil contact surface is determined by the roughness of the pile surface, the type of soil, and the physical–mechanical parameters of the soil^55^; therefore, it is assigned a value of 0.1.

Damping, an inherent property of structures, plays a significant role in structural dynamic analysis. The results of dynamic triaxial experiments on soil indicate that the soil’s damping ratio is not dependent on the excitation frequency; thus, hysteretic damping is commonly employed^56^. In ABAQUS, material damping properties are implemented using Rayleigh damping. Before implementing Rayleigh damping, it is essential to optimize the original Rayleigh damping coefficient formulas (Eqs. (8–10)) to align with hysteretic damping.

25 $$C = \alpha M + \beta K$$

26 $$\alpha = \frac{{2\omega_{i} \omega_{j} \left( {\omega_{j} \zeta_{i} - \omega_{i} \zeta_{j} } \right)}}{{\omega_{i}^{2} - \omega_{j}^{2} }}$$

27 $$\beta = \frac{{2\left( {\omega_{i} \zeta_{i} - \omega_{j} \zeta_{j} } \right)}}{{\omega_{i}^{2} - \omega_{j}^{2} }}$$

where $M$, $K$ are the mass matrix and stiffness matrix, respectively; $\alpha$, $\beta$ are the mass parameter and stiffness parameter, respectively. The mass and stiffness parameters can be determined by Eqs. (17) and (18), where $\zeta_{i}$, $\zeta_{j}$ and $\omega_{i}$, $\omega_{j}$ are the damping ratio and angular frequency of the *i*, and *j* modes, respectively. The equivalent Rayleigh damping coefficient optimization equation under hysteretic damping developed by Cheng^57^ is used in this model:

28 $$\left[ {\begin{array}{*{20}c} {2G} & {\Omega_{1} } \\ {\Omega_{1} } & 0 \\ \end{array} } \right]\left\{ {\begin{array}{*{20}c} A \\ \lambda \\ \end{array} } \right\} = \left\{ {\begin{array}{*{20}c} {2R} \\ {\eta /2} \\ \end{array} } \right\}$$

where $G = \Omega^{{\text{T}}} W\Omega$; $R = \Omega^{{\text{T}}} WY$; $\Omega_{1} = \left\{ {\frac{1}{{2\omega_{1} }}\frac{{\omega_{1} }}{2}} \right\}$; $$\Omega = \frac{1}{2}\left[ {\begin{array}{*{20}c} {1/\omega_{1} } & {\omega_{1} } \\ \begin{gathered} 1/\omega_{2} \\ \cdots \\ 1/\omega_{N} \\ \end{gathered} & \begin{gathered} \omega_{2} \\ \cdots \\ \omega_{N} \\ \end{gathered} \\ \end{array} } \right]$$; $$A = \left\{ {\begin{array}{*{20}c} \alpha & \beta \\ \end{array} } \right\}^{{\text{T}}}$$; $Y = \left\{ {\frac{\eta }{2},\frac{\eta }{2}, \cdots ,\frac{\eta }{2}} \right\}^{{\text{T}}}$; $W = {\text{diag}}\left\{ {w_{k1} ,w_{k2} , \ldots ,w_{kN} } \right\}$; $w_{kN} = \phi_{kn}^{2} \gamma_{n}^{2} S_{a}^{\prime 2}$; $S_{a}^{\prime }$ is the derivative of the acceleration response spectrum; $\eta$ is the hysteresis damping ratio.

#### Application of viscous-spring artificial boundaries and equivalent seismic nodal forces

Based on the viscous-spring artificial boundary conditions and the fluctuation input method as implemented through the wave field decomposition method outlined in Sect.  “Methodology”, it is clear that implementing viscous-spring artificial boundaries and applying equivalent seismic loads in finite element simulations poses significant complexity. To facilitate the construction of viscous-spring artificial boundaries and the application of equivalent seismic nodal forces in finite element software, a Python-based plug-in application^58^ was developed. This application addresses the myriad challenges previously delineated.

**Fig. 6:**
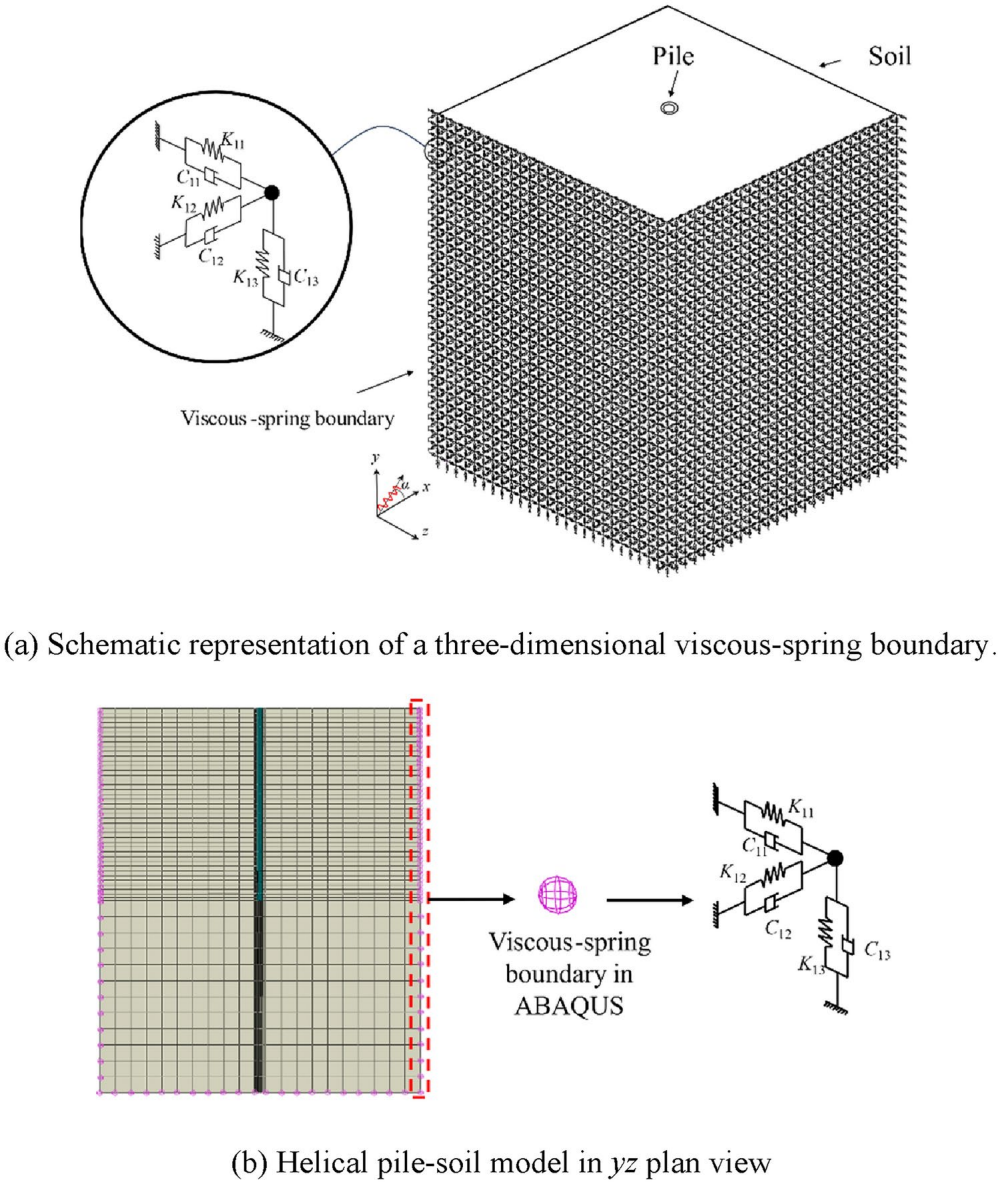
Three-dimensional viscous-spring boundary.

**Fig. 7:**
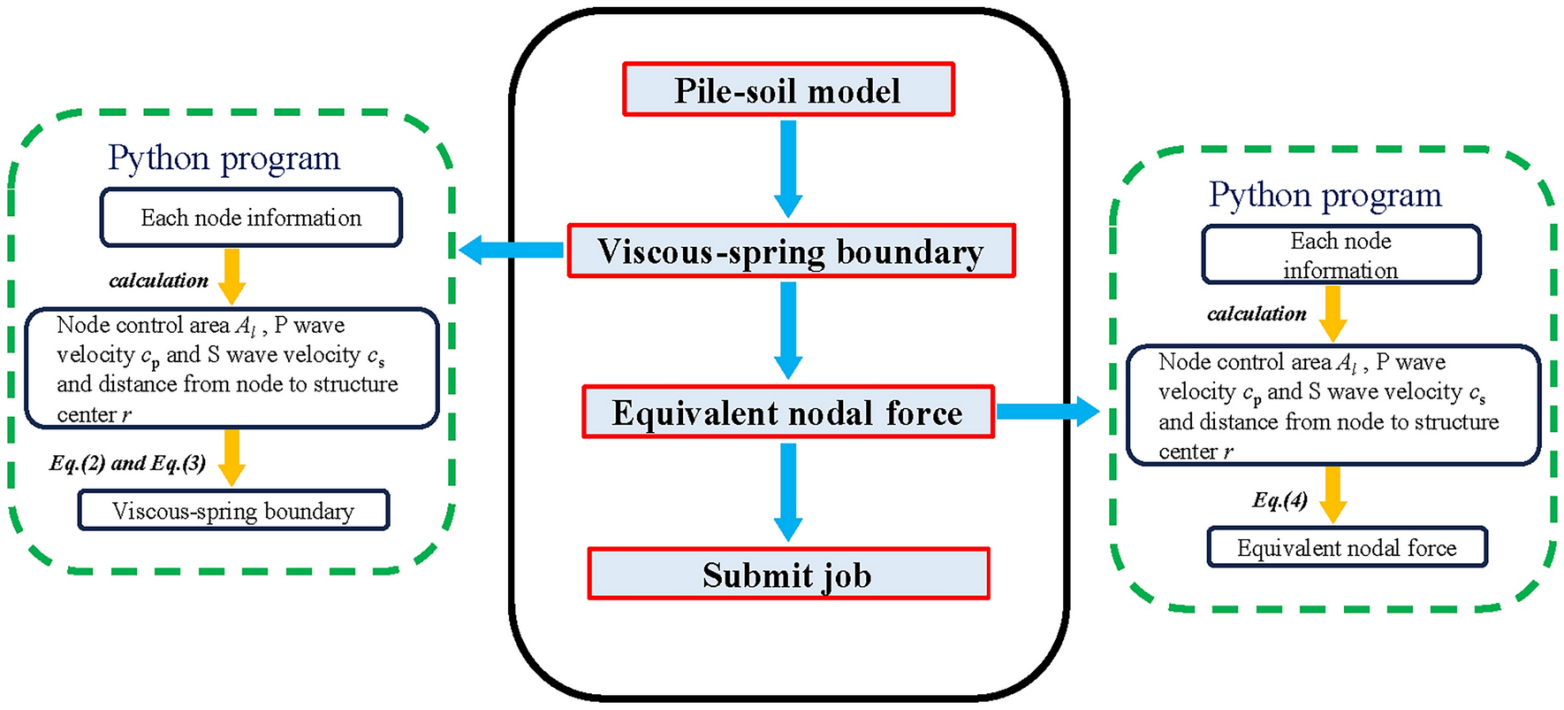
Viscous-spring boundaries and equivalent seismic nodal forces flowchart.

This plug-in application allows for the acquisition of model boundary node numbers, *x*-coordinates, *y*-coordinates, *z*-coordinates, nodal faces, distances from the nodes to the structure’s center, and the nodes’ control areas. Subsequently, this data is systematically organized into an Excel spreadsheet file. Subsequently, by employing this plug-in application with the aforementioned data, the viscous-spring artificial boundary is constructed, as illustrated in Fig. 6. Equivalent seismic nodal forces are entered using the same Excel file, and the displacement and velocity time history curves of the seismic waves are stored in the corresponding working directory. Ultimately, the plug-in applies the required equivalent seismic stresses to the soil boundary nodes. The implementation of viscous-spring artificial boundaries and equivalent seismic nodal forces within the ABAQUS numerical simulation software is carried out through a Python-compiled program, as depicted in Fig. 7.

#### Static-dynamic boundary transformation (establishment of the analysis step)

When conducting dynamic analyses of underground structural models, it is initially assumed that the structure and soil possess initial stresses and displacements resulting from mass and soil pressure. To accurately simulate seismic behavior in practical scenarios, conducting an analysis that integrates both static and dynamic aspects of the helical pile-soil model is crucial. For transitioning from static to dynamic analysis, several common methodologies exist for processing artificial boundaries. This study adopts the first method due to its straightforward concept and ease of implementation in standard numerical software.

Initially, a static ground stress equilibrium analysis step is established within the model, allowing for the calculation of the stress field and soil displacement under gravitational forces. Subsequently, the calculated soil stress is redefined as the initial stress. This process concludes when the vertical displacement of the soil does not exceed 10^–4^ m. Once this criterion is met, the soil’s stress and displacement data are extracted. Following this, a dynamic analysis step is formulated, wherein the fixed displacement boundary conditions are substituted with viscous-spring artificial boundaries. Furthermore, initial stresses are introduced using the keyword “*initial conditions”, ensuring the imposition of initial conditions for dynamic calculations, which is crucial to prevent unrealistic outcomes in the numerical model. Equivalent seismic nodal forces are then applied to each node following the methods outlined in Sect. “Effect of different angles of incidence”.

#### Seismic wave

**Fig. 8:**
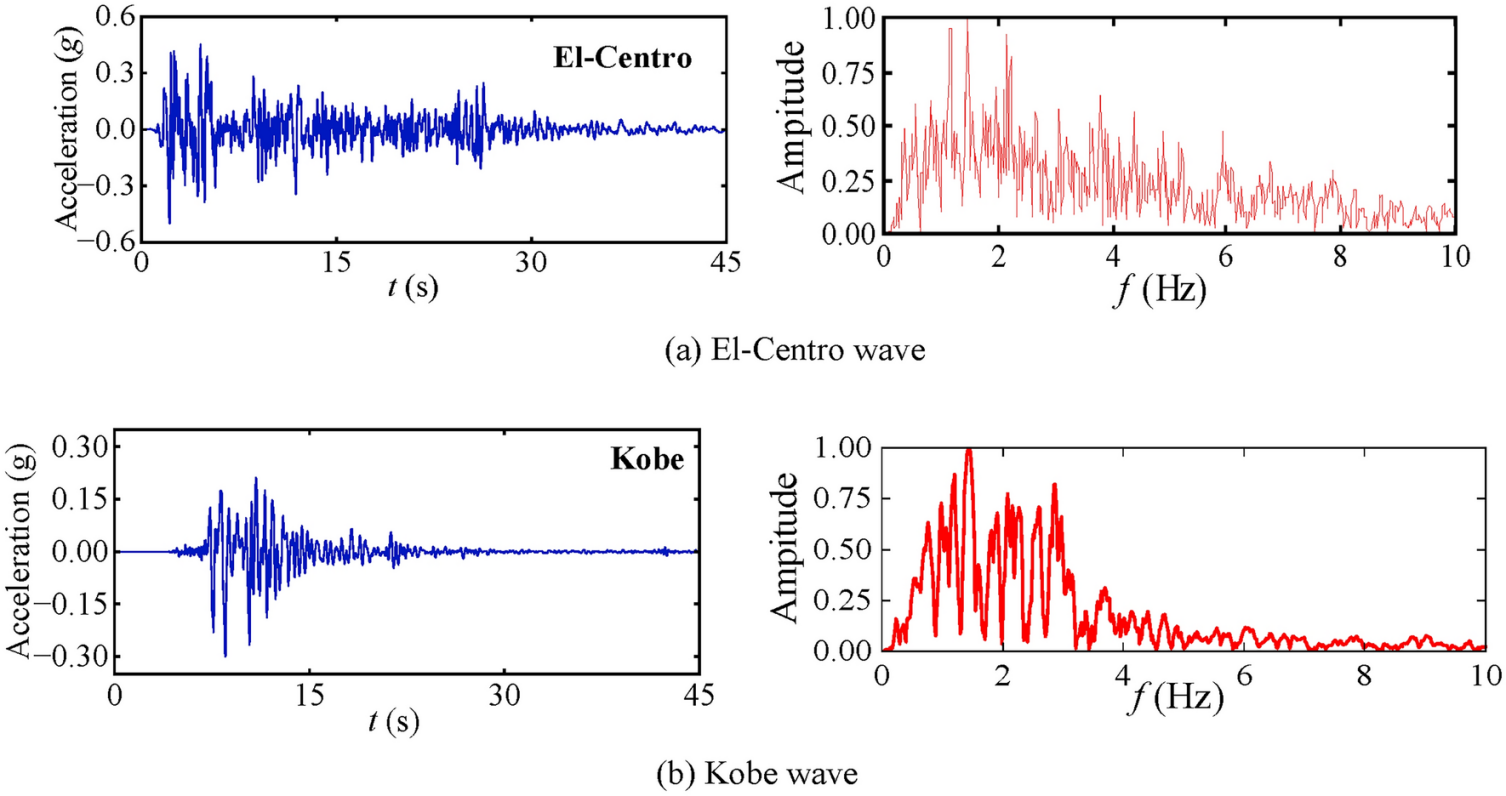
El-Centro and Kobe wave acceleration time history curves and Fourier spectral curves.

**Fig. 9:**
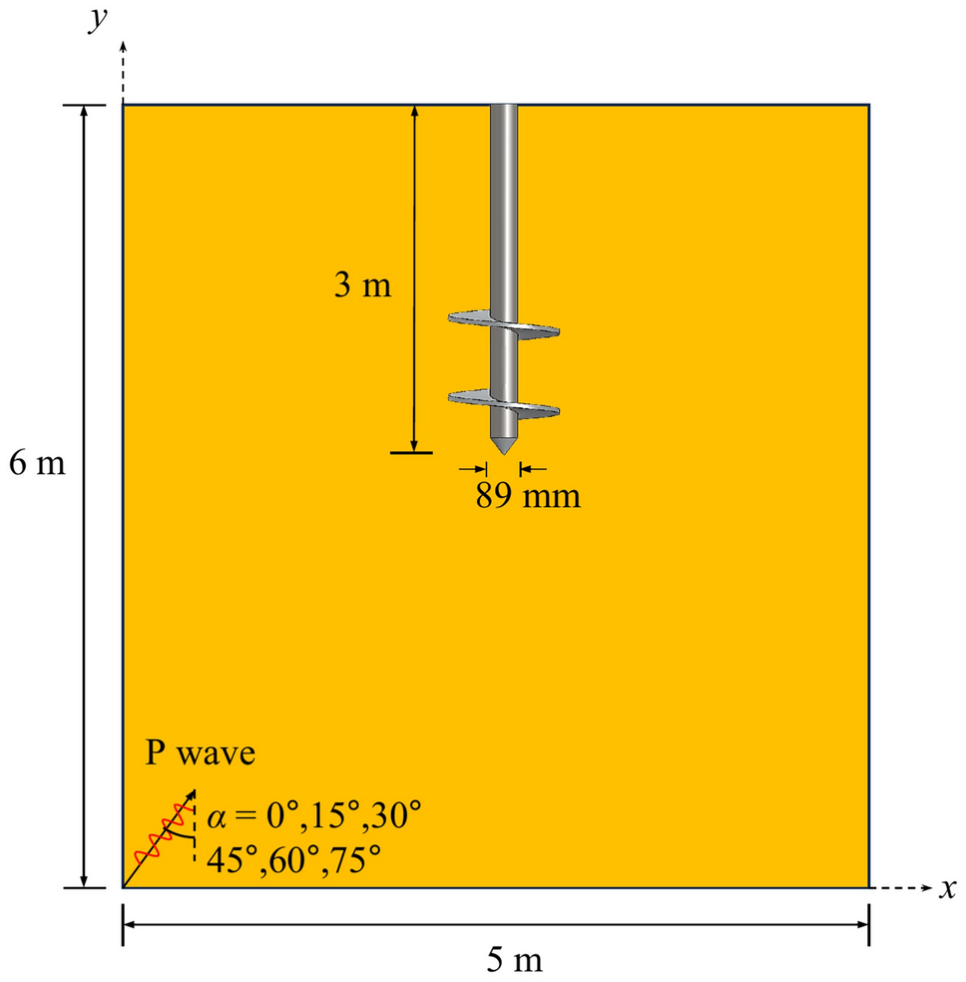
Schematic of model of incident angle.

Two distinct seismic waves, namely the El-Centro and Kobe waves, have been selected for this study to simulate the seismic response of helical piles across a spectrum of seismic frequencies. The peak accelerations of the two seismic waves were adjusted to 0.05 g, 0.1 g, and 0.3 g, respectively, to accurately simulate the seismic subsidence and *p-y* curve responses of the helical piles under varying *PGA*s. The acceleration time history curves and the corresponding Fourier spectra of the two seismic waves are presented in Fig. 8. The frequency range of the El-Centro wave is approximately 0.5 Hz, while that of the Kobe wave is approximately 1 Hz. Figure 9 illustrates the method used to represent the P-wave incidence angle in the x–y plane within the helical pile-soil model. To investigate the impact of various incidence angles on the seismic response of helical piles, six distinct angles were considered: 0°, 15°, 30°, 45°, 60°, and 75°.

As outlined in the previous section, the influence of seismic subsidence and dynamic *p-y* curves on helical piles was investigated, considering various factors such as incidence angles, seismic waves, the number of helical blades, and *PGA*s in soft ground. Table 3 displays the simulated cases, with six different incidence angles (0°, 15°, 30°, 45°, 60°, and 75°) applied across cases 1 through 5, resulting in a total of 30 models being computed.

**Table 3:** Helical pile-soil model characteristics.

Condition	Seismic wave	Number of helical blades	Acceleration amplitude/*g*	Superstructural forces/kN
1	El-Centro	2	0.05	10
2	El-Centro	2	0.1	10
3	El-Centro	2	0.3	10
4	El-Centro	1	0.1	10
5	Kobe	2	0.1	10

## Results and analyses

In this section, the effects of incident angle, seismic wave frequency and *PGA* on the seismic response of helical piles are investigated by comparing the vertical displacement and dynamic *p-y* curves of helical piles-soil under different operating conditions.

### Vertical displacement of helical piles-soil

#### Effects of different seismic waves

**Fig. 10:**
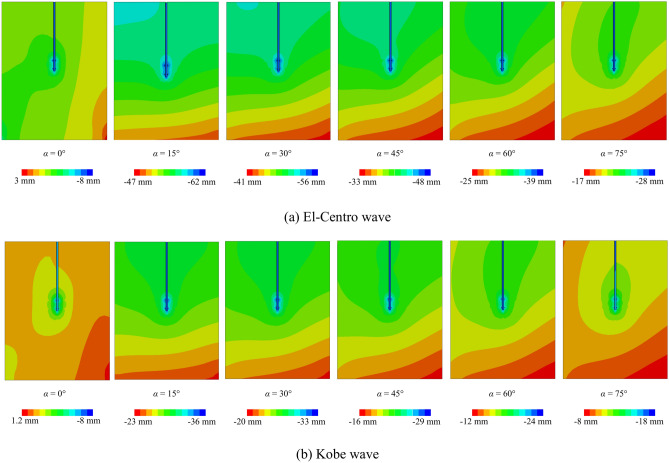
Effect of different seismic waves on vertical displacement of helical pile-soil.

Figure 10 depicts the vertical displacement within the *xy* cross-section of the double-helical blade pile-soil model subjected to a variety of seismic waves. The El-Centro wave is observed to induce greater vertical displacement in the pile-soil model compared to the Kobe wave. Due to the differing frequencies of the seismic waves, the helical pile-soil model exhibits a heightened sensitivity to the El-Centro wave. Furthermore, the displacement cloud of the model shows a predominance of the blue spectrum under El-Centro waves, while a red spectrum is more pronounced when subjected to Kobe waves. This indicates that under El-Centro waves, numerous regions experience greater vertical displacements compared to those under Kobe waves. Therefore, considering the impact of seismic wave frequencies on the vertical displacement of the helical pile-soil model is essential.

### Effect of different numbers of helical blades

**Fig. 11:**
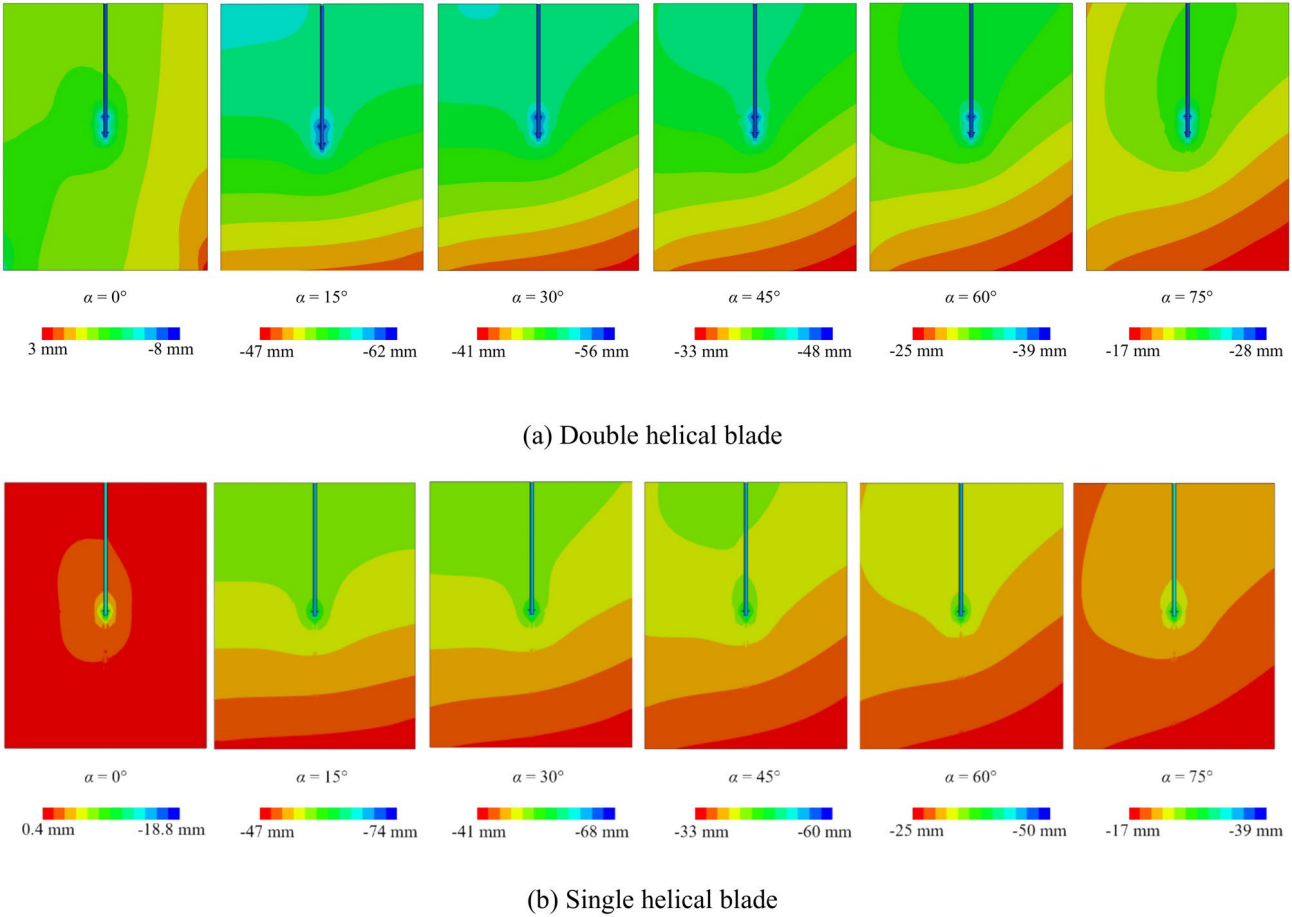
Effect of different numbers of helical blades on vertical displacement of helical pile-soil.

Figure 11 illustrates the vertical displacement response of pile-soil models with varying numbers of helical blades. The single-blade helical pile-soil model demonstrates greater vertical displacement compared to the two-blade model. Additionally, for both the single and double-blade helical pile models, vertical displacement increases from a minimum at 0° to a maximum at 15°, followed by a subsequent decrease. Importantly, the rate of vertical displacement change remains consistent across both single and double-blade models. This suggests that the vertical displacement rate of change in helical piles is unaffected by the number of blades when the incident angle varies. Consequently, the smaller contact area of the single-blade helical pile with the soil, in comparison to the double-blade model, results in a smaller influence area, thereby leading to increased vertical displacement.

### Effect of different angles of incidence

**Fig. 12:**
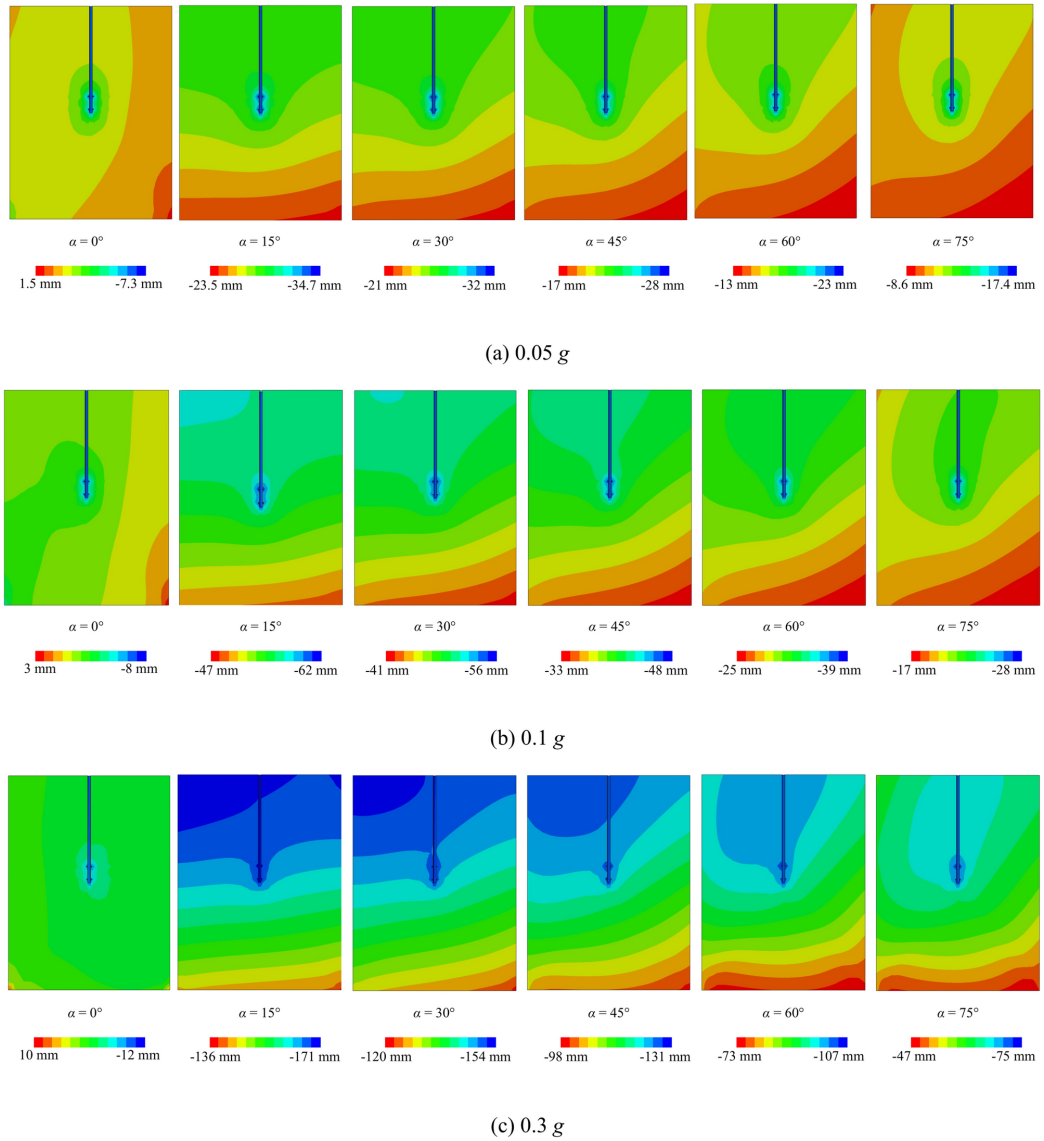
Effect of different incident angles on vertical displacement of helical piles.

Figure 12 depicts the vertical displacement response of the two-blade pile-soil model at various incident angles. It is observed that the vertical displacement of the helical pile-soil model varies with changes in the incident angle. This is similar to the results of literature^7^. At an incident angle of 0°, the seismic wave’s effect on vertical displacement is minimal. Conversely, at an incident angle of 15°, the vertical displacement reaches its peak among the six angles studied. Subsequently, as the incident angle increases, vertical displacement decreases. This demonstrates that the incident angle significantly impacts pile-soil vertical displacement, underscoring the importance of considering incident angle effects in such studies.

### Dynamic p-y curve of helical pile

#### Pile-soil lateral displacement

**Fig. 13:**
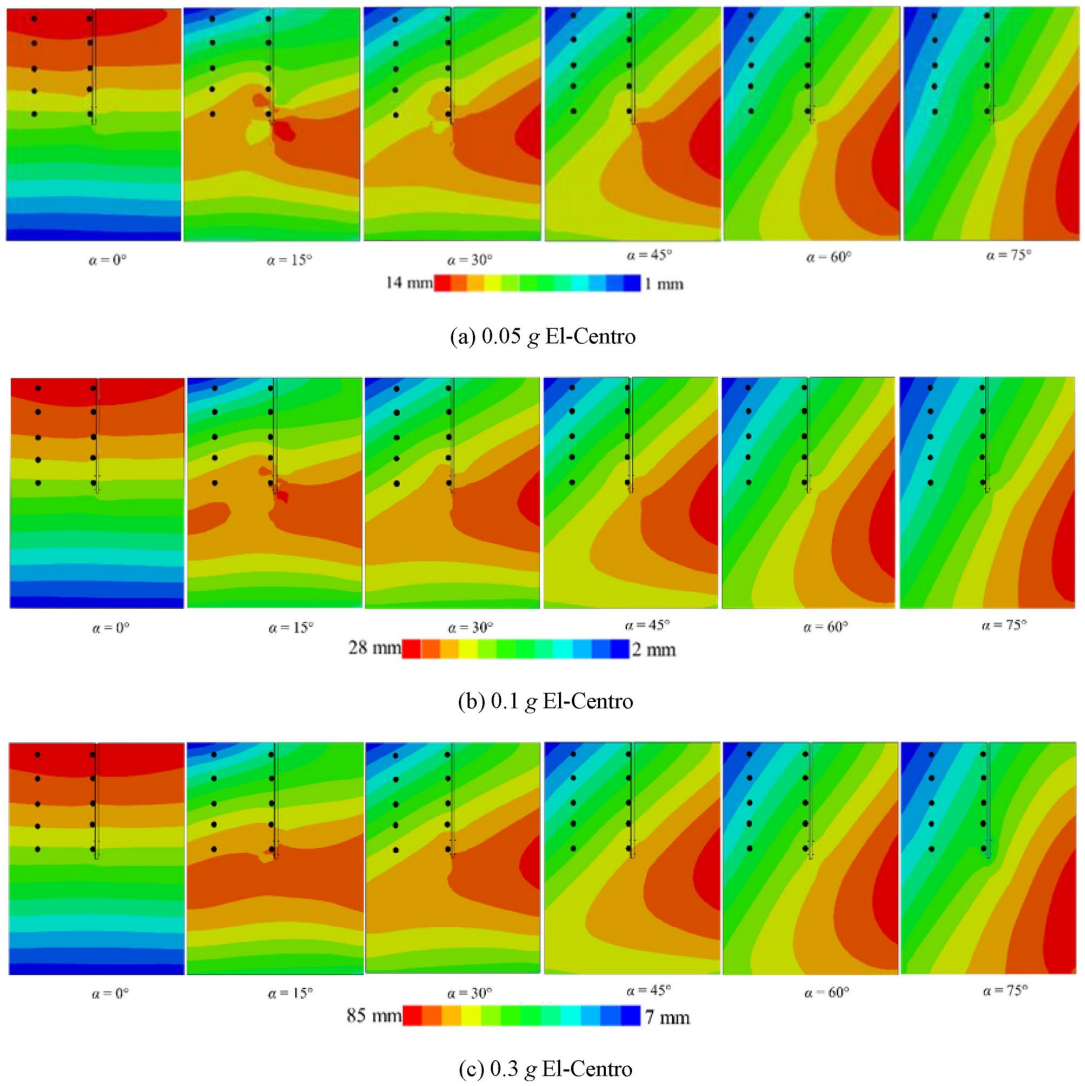
The influence of *PGA* on pile-soil lateral displacement.

**Fig. 14:**
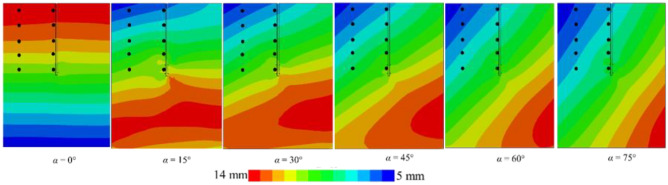
Pile-soil lateral displacement for *PGA* = 0.1 *g* (Kobe wave).

Figure 13 illustrates the lateral displacement cloud of the *xy* cross-section for the pile-soil model under different working conditions. Black dots in the figure indicate the lateral displacements at specific points for both the far-field soil and the pile side. Figure 13a–c depict El-Centro waves with *PGA*s of 0.05 g, 0.1 g, and 0.3 g, respectively, while Fig. 14 presents a Kobe wave with a *PGA* of 0.1 g. The figure reveals that lateral displacements in the pile-soil model vary with changes in the incident angle. At a *PGA* of 0.1 g and incidence angles of 75° and 60°, representative points near the soil surface exhibit a difference of four displacement gradations, each indicated by a distinct color. Conversely, at incidence angles of 45°, 30°, and 15°, representative points near the soil surface differ by three displacement gradations. Finally, at an incidence angle of 0°, displacements near the soil surface remain within a singular displacement gradation. This phenomenon is consistent across representative points at various locations. However, with increasing depth, the effect of the incident angle on lateral displacement decreases for both the pile side and far-field soil. This implies that the impact of incidence angles on relative pile-soil displacement intensifies with shallower depths. Therefore, it is essential to consider the incident angle’s influence on relative displacement between the pile and soil for dynamic *p-y* curve analysis.

**Fig. 15:**
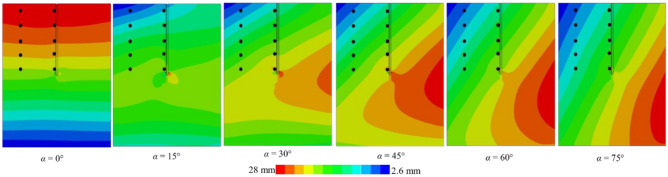
Double helical blade for *PGA* = 0.1 g (El-Centro wave).

Additionally, Fig. 13a reveals a maximum displacement of 14 mm and a minimum of 1 mm. Furthermore, double displacement in Fig. 13b, while it triples in Fig. 13c, aligning with changes in *PGA*. Additionally, the maximum displacement in Fig. 14 decreases to 14 mm, representing a halving of the value, while the minimum displacement increases. Therefore, the effects of *PGA* and seismic wave frequency on lateral displacement in the helical pile-soil model are significant and should not be overlooked. At *PGA*s of 0.1 g and 0.05 g with incident angles of 15° and 45°, lateral displacement increases at the upper left of the second blade and the lower right of the first blade, while it decreases at the upper right of the second blade and the lower left of the first blade, resulting in a displacement lag. This displacement lag phenomenon vanishes at incident angles of 60° and 75°, following an increase in *PGA*. This is attributed to the reduced seismic force area with increased incident angles and *PGA*, necessitating a larger surface area. In comparison to Fig. 15, it can be suggested that the double helical pile more effectively resists oblique incidence and magnitude effects within certain angle ranges and under weak to moderate seismic conditions, but its resistance diminishes with further increases in incidence angle or *PGA*.

### Effects of different seismic waves

**Fig. 16:**
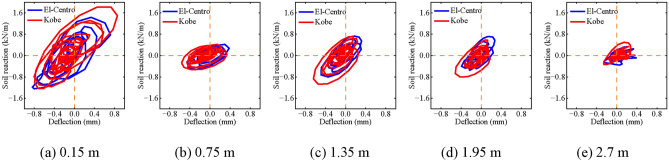
Effect of seismic frequency on dynamic *p-y* curves of helical piles.

Figure 16 illustrates the impact of varying seismic wave frequencies on dynamic *p-y* curves. The hysteresis area of the dynamic *p-y* curve for the El-Centro wave is observed to be smaller than that for the Kobe wave. Furthermore, this area diminishes with increasing depth, indicating the hysteresis area of the dynamic *p-y* curve is minimally influenced by seismic wave frequency. The dynamic *p-y* curve’s backbone further reveals the stiffness properties of the pile-soil model. The backbones of the dynamic *p-y* curves, regardless of seismic wave, do not notably diminish with increased depth, and they appear identical across both curves. This implies that due to the similarity in the main frequencies of the two seismic waves, differing frequencies have a minimal impact on the helical piles’ dynamic *p-y* curve backbones. Echoing Tang’s findings^46^, the Kobe wave induces greater dynamic soil stiffness than the El-Centro wave at various depths, yet this increase in stiffness is not significant between the two waves due to their similar frequencies.

### Effect of different PGAs

**Fig. 17:**
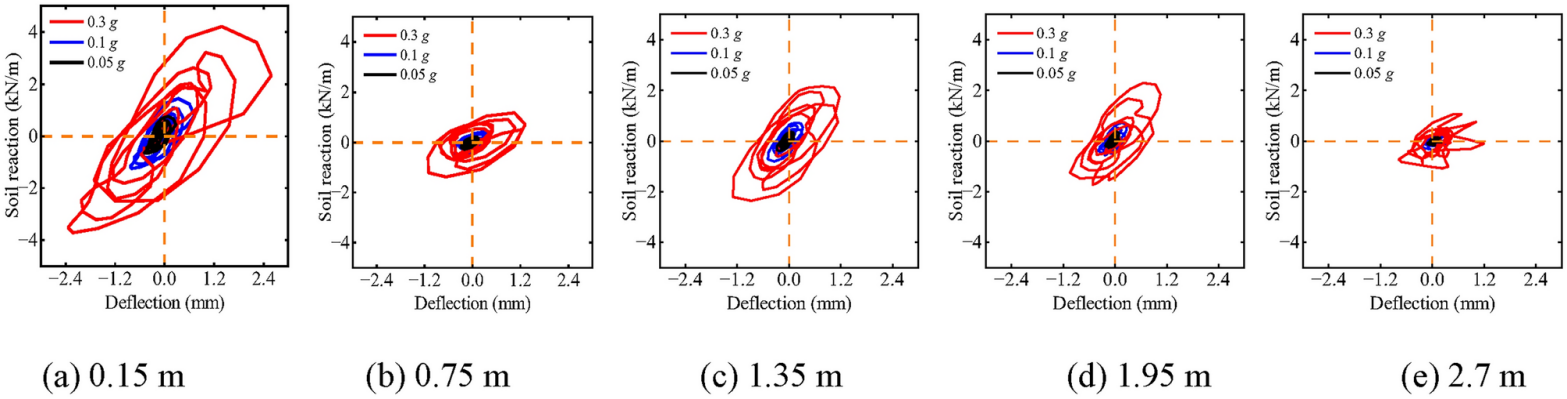
Effect of *PGA*s on dynamic *p-y* curves of helical piles.

Figure 17 illustrates the impact of varying *PGA*s on the dynamic *p-y* curve of helical piles. At constant depth, an increase in *PGA* leads to heightened soil reaction and greater pile-soil relative displacement, resulting in a more pronounced hysteresis curve. This means that as *PGA* increases, power dissipation in helical pile-soil interaction becomes increasingly evident, contributing to enhanced nonlinear behavior. For a given *PGA*, curve areas decrease with increasing depth; furthermore, dynamic *p-y* curves at *PGA*s of 0.1 g and 0.05 g conform more closely to linear interaction than those at 0.3 g. Notably, at a depth of 2.7 m, the hysteresis regions of the *p-y* curves for *PGA*s of 0.1 g and 0.05 g are comparable. However, the difference between *PGA*s of 0.1 g and 0.3 g, a threefold increase, results in more distinct hysteresis areas, especially at the soil surface. This may be due to the pile-soil dynamic properties not showing significant nonlinearity in moderate and mild earthquakes (0.1 g and 0.05 g, respectively), whereas significant nonlinearity is observed during severe earthquakes (0.3 g). Therefore, when analyzing helical pile-soil interactions, especially during strong earthquakes, nonlinear properties must be considered. Furthermore, as *PGA* increases, the backbone slope of the *p-y* curve decreases, indicating a progressive reduction in pile-soil system stiffness.

### Effect of different numbers of helical blades in helical piles

**Fig. 18:**
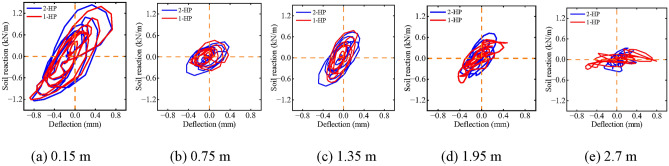
Effect of different numbers of helical blades on dynamic *p-y* curve of helical pile.

Figure 18 illustrates the impact of varying numbers of helical blades on the dynamic *p-y* curve of helical piles. At depths of 0.15 m, 0.75 m, 1.35 m, and 1.95 m, the dynamic *p-y* curves for both double and single helical blades exhibit similar hysteresis areas and backbone slopes. This suggests that the second helical blade has a minimal impact at these depths, due to their distance from the blade. At a depth of 2.7 m, the pile-soil relative displacement for the single-blade helical pile significantly exceeds that of the double-blade pile, with differing backbone slopes observed between the two. However, with increasing depth, the influence of the second helical blade becomes more pronounced. The dynamic *p-y* curve’s backbone is unaffected by the second helical blade at distances far from the blade, yet is significantly influenced at locations closer to it.

### Effect of different angles of incidence

**Fig. 19:**
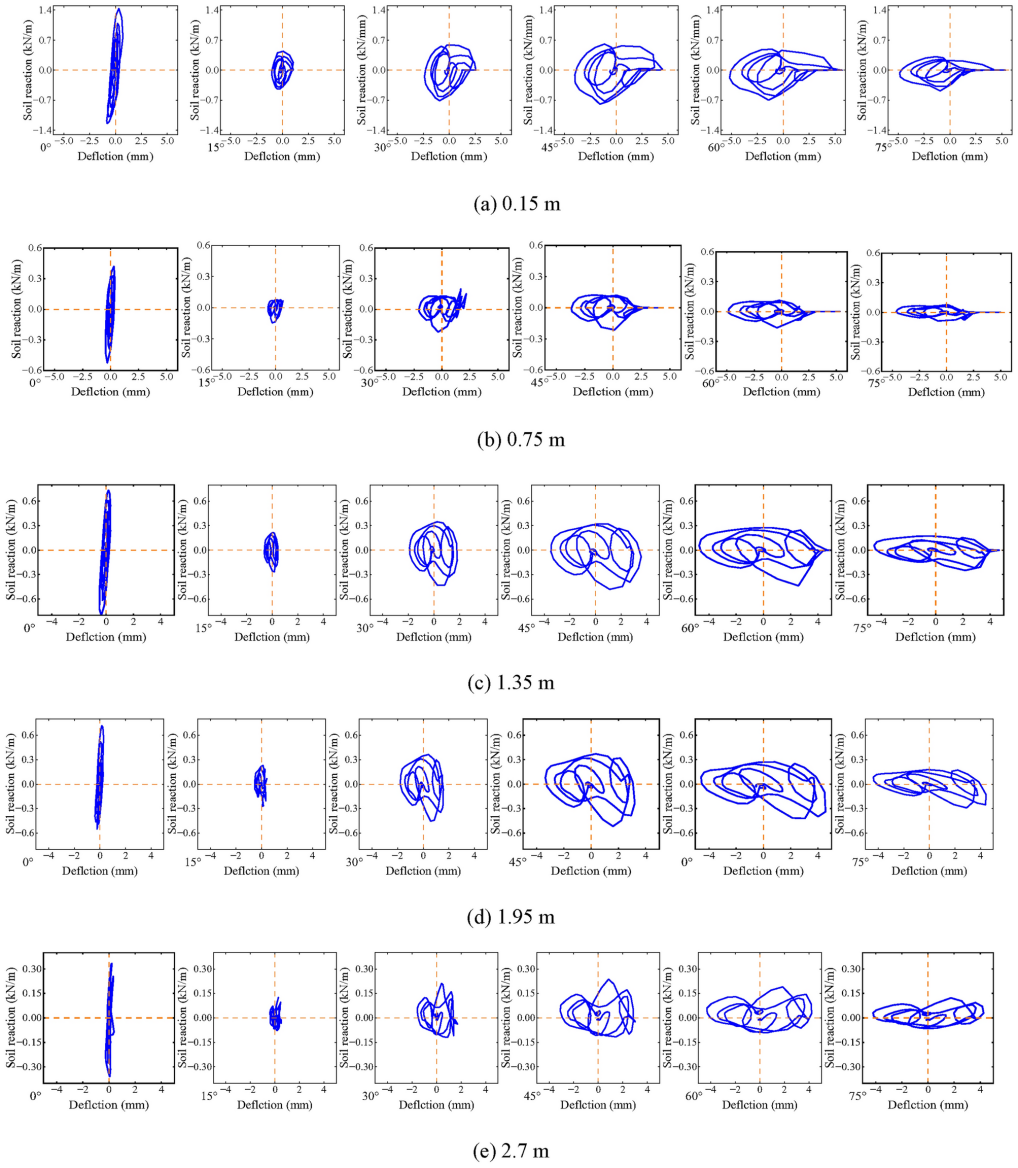
Effect of different incident angles on dynamic *p-y* curves of double helical piles.

Figure 19 illustrates the impact of varying incident angles on the dynamic *p-y* curve of helical piles. The hysteresis area of the dynamic *p-y* curve increases as the incident angle rises, while maintaining a constant depth. The dynamic *p-y* curve’s backbone is most pronounced at an incident angle of 0°. Moreover, the curve’s backbone sharply diminishes at an incident angle of 15°. Subsequent to this, no significant increase in the backbone of the curve occurs at other incident angles, softening the helical pile-soil system’s dynamic stiffness due to a gradual decrease in soil strength.

Additionally, for incident angles of 45°, 60°, and 75°, the soil reaction on the pile side diminishes with increasing incident angle. It is notable, however, that relative displacement between the pile and soil increases under these conditions. This observation implies a decrease in soil strength, resulting in reduced soil capacity to confine the pile. This finding is consistent with Sect. “Correction of dynamic *p*-*y* curves of helical piles in API specification based on the incidence angle”, indicating a significant impact of incident angle on pile-soil relative displacement. Consequently, reduced displacement narrows the interaction area between pile and soil, further limiting the soil’s confining capacity.

## Discussion

### Seismic subsidence effect of helical piles

**Fig. 20:**
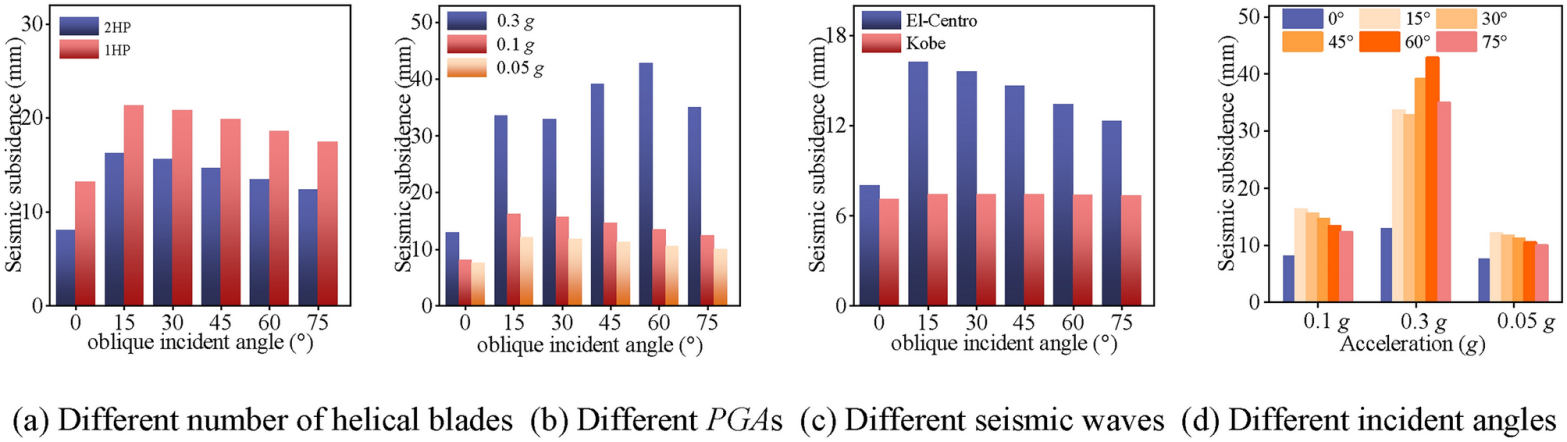
Seismic subsidence values of helical piles under different working conditions.

Sect. “Vertical displacement of helical piles-soil” describes the vertical (absolute) displacements of the helical pile-soil model under various conditions, while this section focuses on seismic subsidence (relative displacements) of helical piles. Figure 20 presents seismic subsidence values of helical piles under varied conditions. Figure 20a shows that multi-blade helical piles experience less seismic subsidence than single-blade piles, which can be attributed to their larger contact area. Similarly, Fig. 20b indicates that, at the same incident angle, a helical pile at 0.1 g exhibits more seismic subsidence than at *PGA* = 0.05 g, by 6.1%. Conversely, at 0.1 g, seismic subsidence is 41% lower than at *PGA* = 0.3 g. This suggests seismic subsidence is negligible at low to medium *PGA*s but significantly increases at higher magnitudes. Thus, this phenomenon warrants careful consideration in high-intensity seismic zones. Observations from Fig. 20c reveal greater seismic subsidence in helical piles subjected to the El-Centro wave than to other seismic waves. Conversely, the Kobe wave exhibits minimal impact on seismic subsidence across different incident angles. However, seismic subsidence varies with incident angle changes when considering the El-Centro wave, consistent with phenomena described in Sect. “Effects of different seismic waves”. Additionally, Fig. 20d demonstrates that seismic subsidence is related to incident angle under varying *PGA*s. The previous analysis illustrates how *PGA*s influence the seismic subsidence of helical piles. For example, the seismic subsidence value initially rises and then decreases with the increase of the incidence angle at *PGA* = 0.1 g, and the maximum peak of the seismic subsidence of the helical pile occurs at 60° at *PGA* = 0.3 g; the variation trend of the seismic subsidence value of the helical pile is consistent with that at *PGA* = 0.05 g. This analysis indicates that different *PGA*s affect the seismic subsidence values of helical piles.

### Effect of oblique incidence of seismic waves on dynamic p-y curves of helical piles

This section further analyzes the effect of incidence angle on the dynamic *p*-*y* curve of the helical pile. To examine the effect of incident angle, it is essential first to introduce the influence factors of incident angle on soil reaction force $F_{o}$ and pile-soil relative displacement $D_{o}$, thereby characterizing their relationship at the pile side. The influence factor for both soil reaction force and pile-soil relative displacement is calculated as the ratio of their values at the peak of the dynamic *p-y* curve to their respective peaks at an incident angle of 0°. Maintaining all other conditions constant and altering only the angle of incidence, we derive the influence factors for soil reaction force and pile-soil relative displacement under varying pile diameter conditions at marine soft soil sites. These factors are derived from the ratio of their peak values on the dynamic *p-y* curve to their benchmark values at an incidence angle of 0°.

**Fig. 21:**
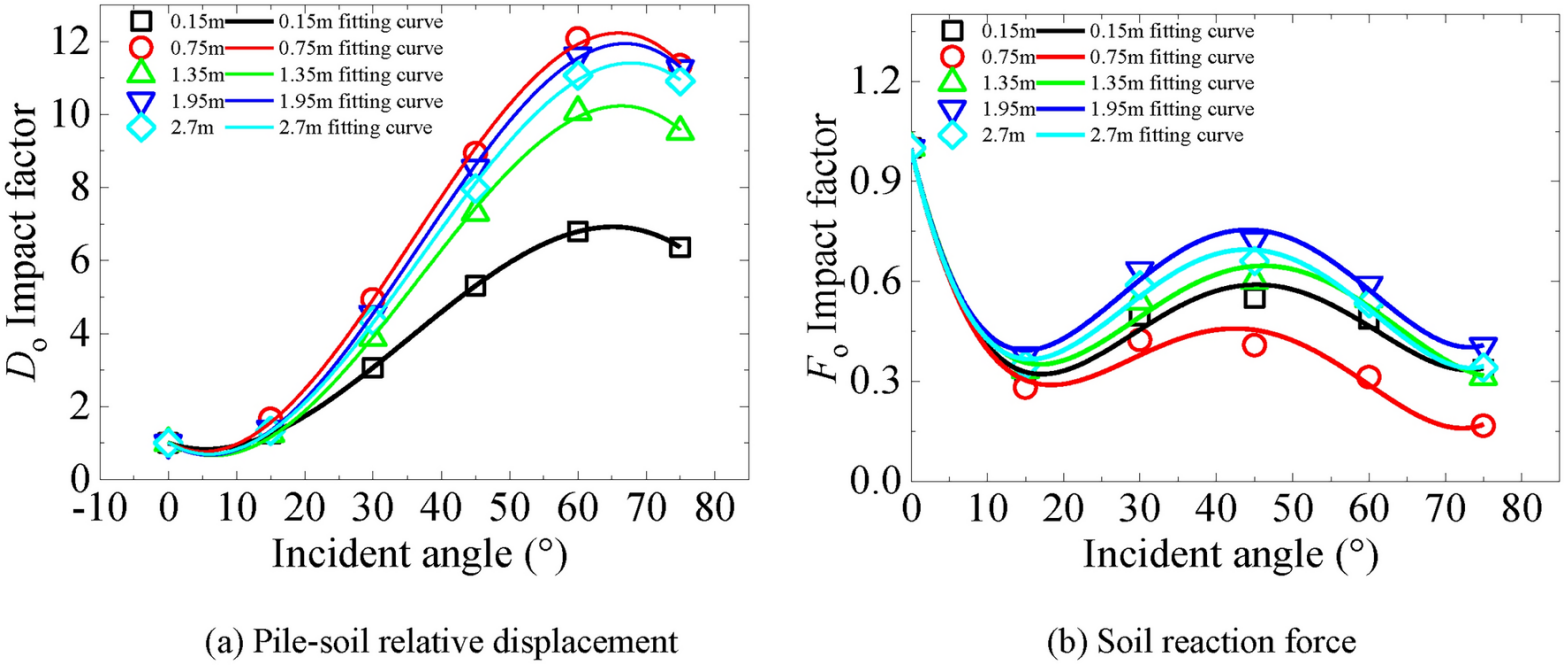
Influence factors of soil reaction force and relative pile-soil displacement at 0.1 g.

Figure 21 depicts the earth reaction force influence factor and displacement influence factor at different incidence angles. It is observed that the displacement influence factor is proportional to the angle. Conversely, the relationship of the earth reaction force influence factor is more complicated, evidencing a tendency to decrease, then increase, and subsequently decrease again. Furthermore, the displacement influence factor attains its maximum value of 12 at a depth of 0.75 m, significantly impacted by the incident angle; similarly, at this depth, the soil reaction force influence factor exhibits the greatest sensitivity to the incident angle. Consequently, the incident angle leads to an increase in the pile-soil relative displacement and a decrease in the soil reaction force at this depth, thereby elucidating the marked changes observed at this depth in the *p-y* curve. Lastly, the fitting curves representing soil reaction force and pile-soil relative displacement influence factors at each depth are detailed in Tables 4 and 5.

**Table 4:** Impact factor fitting curve of pile-soil relative displacement impact factor.

Depth	Pile-soil relative displacement impact factor
0.15 m	$F_{o} = - 0.09727x + 0.00458x^{2} - 7.7616 \times 10^{ - 5} x^{3} + 4.2989 \times 10^{ - 7} x^{4} + 0.99599$
0.75 m	$F_{o} = - 0.09753x + 0.00448x^{2} - 7.68842 \times 10^{ - 5} x^{3} + 4.33416 \times 10^{ - 7} x^{4} + 0.99545$
1.35 m	$F_{o} = - 0.09341x + 0.00439x^{2} - 7.2878 \times 10^{ - 5} x^{3} + 3.91162 \times 10^{ - 7} x^{4} + 0.99555$
1.95 m	$F_{o} = - 0.09704x + 0.005x^{2} - 8.83981 \times 10^{ - 5} x^{3} + 5.01323 \times 10^{ - 7} x^{4} + 0.99702$
2.7 m	$F_{o} = - 0.09841x + 0.00495x^{2} - 8.68691 \times 10^{ - 5} x^{3} + 4.90157 \times 10^{ - 7} x^{4} + 0.9966$

**Table 5:** Impact factor fitting curve of influence factor of soil reaction force.

Depth	Influence factor of soil reaction force
0.15 m	$D_{o} = - 0.06217x + 0.0061x^{2} - 5.75149 \times 10^{ - 4} x^{3} + 1.00243$
0.75 m	$D_{o} = - 0.10525x + 0.01092x^{2} - 1.02327 \times 10^{ - 4} x^{3} + 1.03349$
1.35 m	$D_{o} = - 0.11846x + 0.00984x^{2} - 8.98946 \times 10^{ - 5} x^{3} + 1.03189$
1.95 m	$D_{o} = - 0.1193x + 0.01089x^{2} - 9.95919 \times 10^{ - 5} x^{3} + 1.01738$
2.7 m	$D_{o} = - 0.10932x + 0.01001x^{2} - 9.05362 \times 10^{ - 5} x^{3} + 1.01212$

#### Correction of dynamic p-y curves of helical piles in API specification based on the incidence angle

The analysis of dynamic *p-y* curves for helical piles, as presented in Sect. “Results and analyses” identifies various influencing factors, with the degree of incident angle exerting a significant effect. Typically, soil stiffness and strength emerge as the primary factors influencing the *p-y* curve, with soil stiffness defined as the ratio of soil reaction force to pile displacement. Soil stiffness is crucial during transient motions, as both the reaction force at the pile side and soil deformation are contingent upon the soil’s initial stiffness and deformation, especially when pile-soil displacement is minimal. Conversely, at larger pile-soil displacements, soil strength assumes increased importance, with the soil reaction force being dependent on the ultimate soil reaction force. This aspect is further elucidated in this section through the examination of the API-recommended *p-y* curve.

The dynamic Winkler foundation beam theory, also known as the dynamic *p-y* curve method, represents a primary approach for analyzing pile-soil dynamic interaction, chiefly employing the Matlock model^59,60^. Specifically, the Matlock model, recommended by the API specification, serves as a calculation method for assessing pile-soil interaction in soft ground, as follows:

29 $$p = \left\{ {\begin{array}{*{20}l} {0.5p_{u} \left( {y/y_{50} } \right)^{\frac{1}{3}} } \hfill & {y/y_{50} < 8} \hfill \\ {p_{u} } \hfill & {y/y_{50} \ge 8} \hfill \\ \end{array} } \right.$$

30 $$y_{50} = 2.5\varepsilon_{50} D$$

31 $$p_{u} = \left\{ {\begin{array}{*{20}l} {\left( {3c_{u} + \gamma z + Jc_{u} z/D} \right)D} \hfill & {z < z_{R} } \hfill \\ {9c_{u} D} \hfill & {z \ge z_{R} } \hfill \\ \end{array} } \right.$$

32 $$z_{R} = 6D/\left( {\gamma D/c_{u} + J} \right)$$

where $p_{u}$ is the ultimate soil reaction force of soft clay; $y$ is the lateral displacement; $y_{50}$ is the lateral deformation of the soil around the pile at half of the ultimate resistance; $\varepsilon_{50}$ is the strain at half of the ultimate strength of undrained compression; $z_{R}$ is the depth of the turning point of the ultimate lateral soil resistance; $c_{u}$ is the undrained shear strength of the cohesive soil; $\gamma$ is the mass weight of the soil; and $J$ is an empirical coefficient which is usually taken to be 0.5. From the equation, it is evident that the *p-y* curve for soft clay can be determined through an approximation of the ultimate soil reaction. Additionally, the curve presented in this study aligns more closely with the model at an incident angle of 0°. However, at incident angles other than 0°, the API curve does not align as closely with that of this study. At an incidence angle of 0°, the API-recommended *p-y* curve shows better alignment with the curve derived from this study’s model. However, at incidence angles other than 0°, there is poor agreement between the API curve and the calculated results of this study’s model. Therefore, under the assumption that the ultimate soil reaction for soft clay remains constant, this study adjusts the strain to half of the undrained compressive ultimate strength as specified by the API, and introduces for the first time a soil strain calculation formula applicable to infinite sites^61^.

33 $$\varepsilon = \frac{\partial u(t)}{{\partial t}}\frac{1}{c}$$

where $\varepsilon$ is the free-field strain of the infinite ground, $\partial u(t)/\partial t$ is the incident wave velocity, and $c$ is the S-wave or P-wave velocity. However, in this model, for the semi-infinite ground, the incident wave and reflected wave need to be considered, then the above equation can be changed to:

34 $$\varepsilon = \left( {\frac{\partial u(t)}{{\partial t}} - \frac{\partial u(t - \Delta t)}{{\partial t}}} \right)\frac{1}{c}$$

where $\partial u(t - \Delta t)/\partial t$ for the reflected wave speed; $\Delta t$ for the delay time, because the incident surface is closer to the helical pile model, it can be equated to $\Delta t = 2H/c$, where $H$ for the pile at any point of the burial depth distance; $\Delta t$ for the infinitesimal quantity. Rewrite the formula as:

35 $$\varepsilon = \left( {\frac{\partial u(t)}{{\partial t}} - \frac{\partial u(t - \Delta t)}{{\partial t}}} \right)\frac{1}{c\Delta t}\Delta t = \frac{2H}{{c^{2} }}\left( {\frac{\partial u(t)}{{\partial t}} - \frac{\partial u(t - \Delta t)}{{\partial t}}} \right)\frac{1}{\Delta t} \approx \frac{2H}{{c^{2} }}\frac{{\partial^{2} u(t)}}{{\partial t^{2} }}$$

where $\partial u(t)/\partial t$ is the incident wave acceleration. The first-order derivation of the node velocity equation in Eq. (7) obtains:

36 $$\frac{{\partial^{2} u(t)}}{{\partial t^{2} }} = \frac{{\partial^{2} u_{0} (t - \Delta t_{1} )}}{{\partial t^{2} }}\sin \alpha + B_{1} \frac{{\partial^{2} u_{0} (t - \Delta t_{2} )}}{{\partial t^{2} }}\sin \alpha + B_{2} \frac{{\partial^{2} u_{0} (t - \Delta t_{3} )}}{{\partial t^{2} }}\cos \beta$$

where $\partial^{2} u(t)/\partial t^{2}$ is the initial acceleration, substituting Eq. (36) into Eq. (30) obtains:

37 $$y_{50} = 2.5\frac{2HD}{{c^{2} }}\frac{{\partial^{2} u_{0} (t - \Delta t_{1} )}}{{\partial t^{2} }}\sin \alpha + B_{1} \frac{{\partial^{2} u_{0} (t - \Delta t_{2} )}}{{\partial t^{2} }}\sin \alpha + B_{2} \frac{{\partial^{2} u_{0} (t - \Delta t_{3} )}}{{\partial t^{2} }}\cos \beta$$

The recommended *p-y* curves based on the API specification can be obtained by combining Eqs. (29), (31), (32) and (37) for different incidence angles. This allows a simple prediction of dynamic *p-y* curves at different incidence angles by Eq. (37).

## Conclusions

The study investigates the seismic performance of helical piles by analyzing the effects of incident angle, helical blade count, seismic wave characteristics, and *PGA* on vertical displacement, seismic subsidence, and dynamic* p*-*y* curves. This study employs a viscous-spring boundary helical pile-soil model, from which the following conclusions are derived:

1. Vertical displacement is significantly affected by seismic wave frequency during non-vertical incidence. For helical piles located farther from the epicenter, it is imperative to consider the effects of non-vertical waves across a range of frequencies.
2. *PGA* and incident angle are critical factors influencing vertical displacement, with their combined effects being more pronounced than those of seismic wave frequency. In regions of high seismic intensity, increasing the number of helical blades effectively reduces settlement by enhancing soil-pile interaction.
3. The incident angle has the greatest impact on lateral displacement, especially in proximity to the ground surface. An increased number of helical blades improves stability by diminishing the effects of incident angles through improved soil contact.
4. At higher *PGA*s (e.g., 0.3 g), the dynamic stiffness of the helical pile-soil system decreases notably, as observed in the slope of the *p*-*y* curve backbones. The impact of high *PGA*s leads to enhanced nonlinearity, particularly at depths nearer to the soil surface, where hysteresis effects are most pronounced.
5. The seismic subsidence of helical piles varies significantly under diverse conditions, exhibiting ranges of 23.8%–39.1% depending on the number of blades, 37% to 64% in relation to *PGA*, 11% to 40% attributed to seismic wave frequency, and 10% to 51% influenced by incident angle. These findings underscore the critical role of these parameters in mitigating settlement.

## Shortcomings and prospects

1. The viscous-spring boundary model employed in this study assumes linear soil behavior, thereby simplifying soil-structure interactions. However, in practice, soils often exhibit nonlinear, hysteretic, and plastic behavior under high-intensity seismic loading, which may compromise the reliability of the results. Future studies should account for nonlinear soil behavior and plasticity effects to enhance the reliability of dynamic *p*-*y* curves and seismic settlement assessments.
2. Since the P-wave causes longitudinal deformation of the soil, the soil-pile interaction is more likely to cause vertical deformation and displacement of the pile. Therefore, this study mainly focuses on the oblique incidence of P-waves, but does not explicitly consider the possible coupling effect between P-waves and S-waves in complex seismic wave fields. Future studies should investigate how the combined wave effect affects the pile-soil interaction in the case of oblique incidence.
3. This study assumes that the soil layers are homogeneous, which simplifies the analysis but overlooks variations in heterogeneous soil profiles common in engineering practice. Future models should consider the effects of layered soils to simulate real-world conditions more accurately and to address depth-dependent soil properties.

## Data Availability

The data that supports the findings of this study are available on request from the corresponding author. The data is not publicly available due to privacy or ethical restrictions.
